# Supplementary material for: Sensitive detection of total anti-Spike antibodies and isotype switching in asymptomatic and symptomatic individuals with COVID-19
Source: Cell Rep Med. 2021 Jan 16;2(2):100193. doi: 10.1016/j.xcrm.2021.100193 (PMC7816583; doi:10.1016/j.xcrm.2021.100193)
Supplement: Document S1. Figures S1 and S2 and Tables S1 and S2 [file mmc1.pdf]

Cell Reports Medicine, Volume *2*

Supplemental Information

Sensitive detection of total anti-Spike antibodies and isotype switching in asymptomatic and symptomatic individuals with COVID-19

Yun Shan Goh, Jean-Marc Chavatte, Alicia Lim Jieling, Bernett Lee, Pei Xiang Hor, Siti Naqiah Amrun, Cheryl Yi-Pin Lee, Rhonda Sin-Ling Chee, Bei Wang, Chia Yin Lee, Eve Zhi Xian Ngoh, Cheng-I Wang, Barnaby Edward Young, Paul A. Tambyah, Shirin Kalimuddin, Surinder Pada, Seow-Yen Tan, Louisa Jin Sun, Mark I-Cheng Chen, Yee-Sin Leo, David C. Lye, Lisa F.P. Ng, Raymond Tzer Pin Lin, and Laurent Renia

Sensitive detection of total anti-Spike antibodies and isotype switching in asymptomatic and symptomatic COVID-19 patients

Yun Shan Goh[1,2], Jean-Marc Chavatte[3], Alicia Lim Jieling[3], Bernett Lee[2], Pei Xiang Hor[1,2], Siti Naqiah Amrun[1,2], Cheryl Yi-Pin Lee[1,2], Rhonda Sin-Ling Chee[1,2], Bei Wang[2], Chia Yin Lee[2], Eve Zhi Xian Ngoh[2], Cheng-I Wang[2], Barnaby Edward Young[3,4,5], Paul A. Tambyah[3,6], Shirin Kalimuddin[7,8], Surinder Pada[9], Seow-Yen Tan[10], Louisa Jin Sun[11], Mark I-Cheng Chen[3,12], Yee-Sin Leo[3,4,5,13], David C. Lye[3,4,5,13], Lisa F.P. Ng[1,2,14,15,16,17], Raymond Tzer Pin Lin[3,13,18], Laurent Renia[1,2,17,18,19,*]

[1]Infectious Diseases Laboratories (ID Labs), Agency for Science, Technology and Research (A*STAR), Immunos, Biopolis, Singapore 138648, Singapore.

[2]Singapore Immunology Network, Agency for Science, Technology and Research (A*STAR), Immunos, Biopolis, Singapore 138648, Singapore.

[3]National Centre for Infectious Diseases, 16 Jalan Tan Tock Seng, Singapore 308442, Singapore.

[4]Department of Infectious Diseases, Tan Tock Seng Hospital, 11 Jalan Tan Tock Seng, Singapore 308433, Singapore.

[5]Lee Kong Chian School of Medicine, Nanyang Technological University, 11 Mandalay 20 Road, Singapore 308232, Singapore.

[6]Department of Medicine, National University Hospital, 5 Lower Kent Ridge Road, Singapore 119074, Singapore.

[7]Department of Infectious Diseases, Singapore General Hospital, 31 Third Hospital Ave, Singapore 168753, Singapore.

[8]Emerging Infectious Disease Program, Duke-NUS Medical School, 8 College Road, Singapore 169857, Singapore.

[9]Division of Infectious Diseases, Ng Teng Fong Hospital, 1 Jurong East Street 21, Singapore 609606, Singapore.

[10]Department of Infectious Diseases, Changi General Hospital, 2 Simei Street 3, Singapore 529889, Singapore.

[11]Alexandra Hospital, 378 Alexandra Road, Singapore 159964, Singapore.

[12]Saw Swee Hock School of Public Health, National University of Singapore and National University Health System, 12 Science Drive 2, Singapore 117549, Singapore

[13]Yong Loo Lin School of Medicine, National University of Singapore and National University Health System, 10 Medical Drive, Singapore 117597, Singapore.

[14]Department of Biochemistry, Yong Loo Lin School of Medicine, National University of Singapore, Singapore 117596, Singapore.

[15]National Institute of Health Research, Health Protection Research Unit in Emerging and Zoonotic Infections, University of Liverpool, Liverpool, United Kingdom.

[16]Institute of Infection, Veterinary and Ecological Sciences, University of Liverpool, Liverpool, United Kingdom.

[17]Department of Microbiology and Immunology, Yong Loo Lin School of Medicine, National University of Singapore, Singapore 117597, Singapore.

[18]Senior author

[19]Lead contact

***Corresponding author**: Laurent Renia, A* STAR Infectious Diseases Laboratories (ID Labs), 8A Biomedical Grove, #03-15, Immunos Building, Biopolis, Singapore 138648; Tel: +65 64070005; Fax: +65 6464 2056; Email: renia_laurent@immunol.a-star.edu.sg

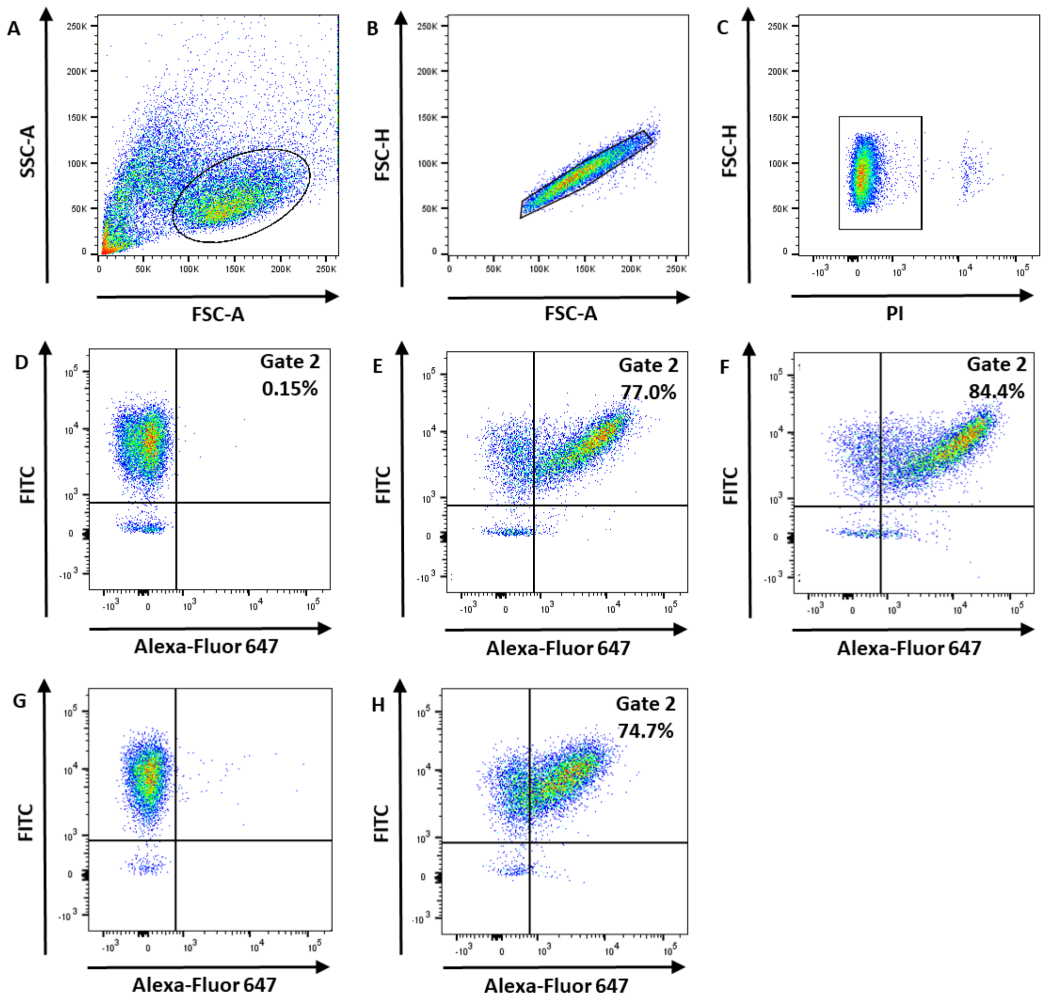

Figure S1. FACS plot analysis. Cells were gated on: (A) FSC-A/SSC-A to exclude cell debris, (B) FSC-A/FSC-H to select for single cells, (C) FSC-A/PI to select for live cells (PI-negative population), (D, E, F, G, H) FITC/Alexa Fluor 647. Binding is determined by the percentage of GFP-positive S protein-expressing cells that are bound by specific antibody, indicated by the events that are Alexa Fluor 647- and FITC-positive (Gate 2). (D) PBS control; (E) 6.25 µg/ml ACE-Human Fc; (F) 1 µg/ml 5A6 S protein RBD-specific monoclonal antibody; (G) healthy control plasma, 1:100 diluted; (H) COVID-19 patient plasma, 1:100 diluted. Related to Figure 1.

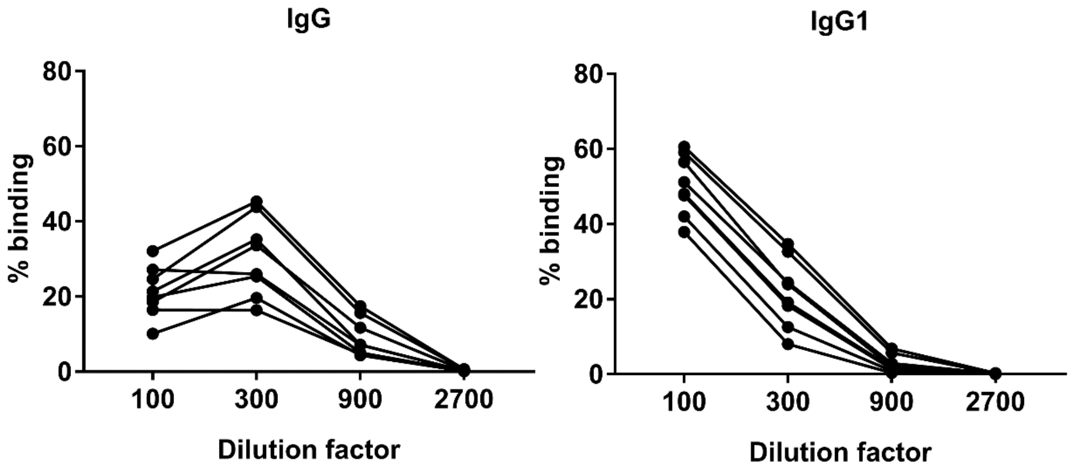

Figure S2. Effect of sample dilution on total IgG and IgG1 response. A total of eight samples from the NPHL cohort, where higher IgG1 response was observed over total IgG at dilution factor 100, were further analysed. The total IgG and IgG1 response were analysed by the SFB assay over four dilution factors (100, 300, 900, 2700). Data are shown as mean of two independent experiments. Related to Figure 4.

Table S1. Demographic and clinical information of symptomatic COVID-19 patients, healthy donors, recovered SARS, seasonal human CoV patients, and samples from National Public Health laboratory (NPHL), Related to STAR Methods Section

**Symptomatic COVID-19 patients**	**Patients (N=81)**
Age, Mean years (SD)	45 (13)
Gender, n (%)	
Male	48 (59.3%)
Female	33 (40.7%)
Ethnicity, n (%)	
Chinese	68 (84.0%)
Others	13 (16.0%)
Co-morbidities, n (%)	
Diabetes	7 (8.6%)
Hypertension	15 (18.5%)
Others	11 (13.6%)
Vital signs at admission	
Temperature, Mean °C, (SD)	37.7 (0.9)
Heart rate, Mean beats/minute (SD)	91.4 (16.6)
Respiratory rate, Mean rate per minute (SD)	18.4 (1.9)
Diastolic blood pressure, Mean mmHg (SD)	97.5 (2.4)
Systolic blood pressure, Mean mmHg (SD)	132.2 (18.5)
Oxygen saturation, Mean % (SD)	77.8 (15.2)
Laboratory findings	
Haemoglobin, Mean g/dL (SD)	13.8 (1.6)
Haematocrit, Mean % (SD)	40.8 (4.6)
Platelets, Mean x 10^9/L (SD)	194.8 (69.8)
White blood cells, Mean x 10^9/L (SD)	5.3 (3.0)
Lymphocytes, Mean x 10^9/L (SD)	1.2 (0.6)
Neutrophils, Mean x 10^9/L (SD)	4.3 (7.7)
Monocytes, Mean x 10^9/L (SD)	0.6 (1.1)
C-reactive protein, Mean mg/L (SD)	37.4 (55.7)
Creatinine, Mean µmol/L (SD)	75.0 (45.3)
Lactate dehydrogenase, Mean U/L (SD)	514.3 (298.4)
Alanine aminotransferase, Mean U/L (SD)	34.6 (28.1)
**Healthy donors**	**N=22***
Age, Mean years (SD)	45 (13)
Gender, n (%)	
Male	9 (40.9%)
Female	12 (54.5%)
**Recovered SARS**	**N=20**
Age, Mean years (SD)	48 (13)
Gender, n (%)	
Male	5 (25.0%)
Female	15 (75.0%)
**Seasonal human CoV**	**N=20**
Age, Mean years (SD)	44 (16)
Gender, n (%)	
Male	7 (53.8%)
Female	6 (46.2%)
**NPHL Samples**	**N=108**
**PCR-positive**	
**Symptomatic**	**16**
Convalescent COVID-19 patients	14/16

Random testing of general population	2/16
**No symptom**	**34**
MOH** surveillance of dormitory residents	33/34
MOH surveillance of suspected and quarantined cases	1/34
**Symptom-unknown**	**11**
MOH surveillance of suspected and quarantined cases	11/11
**PCR-negative**	
**Symptom-unknown**	**13**
MOH surveillance of suspected and quarantined cases	13/13
**PCR-negative/not done**	
**No Symptom**	**20**
MOH surveillance of dormitory residents	20/20
**PCR-unknown**	
**Symptom-unknown**	**15**
MOH surveillance of suspected and quarantined cases	9/15
Random testing of general population	6/15

* Information of one donor unknown. **Ministry of Health. SARS: Severe Acute Respiratory Syndrome Coronavirus; human CoV: human coronavirus

Table S2. DNA sequence of codon-optimised SARS-CoV-2 S gene and primers used to sequence full length SARS-CoV-2-S protein, Related to STAR Methods Section

SARS-CoV-2 S gene Codon-optimised	ATGTTTGTATTCTTGGTACTTCTCCCATTGGTATCTTCTCAATGCGTTAACCTTACCACACGC<br>ACCCAACTGCCCCCGGCCTACACTAATAGCTTTACGCGGGGTGTCTACTATCCCGACAAAGT<br>CTTTCGATCCAGTGTGCTCCACTCCACCCAGGATCTTTTCCTTCCCTTTTTTTCTAATGTTAC<br>GTGGTTCCACGCAATCCATGTATCCGGTACGAATGGGACAAAACGCTTTGACAATCCAGTG<br>CTGCCATTTAATGATGGAGTGTACTTTGCATCTACCGAGAAGAGTAACATCATCAGAGGAT<br>GGATCTTCGGAACGACCTTGGACTCCAAAACGCAATCCTTGCTTATCGTTAACAATGCAAC<br>GAATGTTGTCATCAAAGTTTGCGAATTCCAATTCTGTAACGATCCCTTCCTCGGTGTTTATTA<br>TCATAAAAATAATAAATCTTGGATGGAAAGTGAGTTCCGCGTATACAGTTCCGCCAATAAT<br>TGTACCTTCGAATACGTAAGTCAACCGTTCTTGATGGATCTGGAAGGTAAACAGGGTAACT<br>TTAAGAACCTTCGGGAGTTTGTTTTTAAGAACATAGACGGCTACTTTAAGATCTATAGTAAA<br>CATACGCCAATTAACTTGGTTAGAGATCTCCCGCAGGGGTTTTCAGCATTGGAGCCGCTCGT<br>CGACCTCCCCATAGGTATAAATATAACTCGGTTTCAAACACTGCTGGCGCTCCACCGCAGCT<br>ACCTGACGCCTGGGGATTCTTCTTCCGGTTGGACTGCAGGCGCTGCTGCATATTATGTAGGG<br>TACCTGCAACCGAGAACCTTTCTCCTTAAGTACAACGAGAATGGCACTATTACGGACGCTG<br>TCGATTGTGCACTCGACCCCTTGAGTGAGACGAAGTGTACACTGAAAAGCTTTACTGTTGA<br>AAAGGGAATATATCAGACATCCAACTTTAGAGTTCAGCCAACAGAATCCATCGTTCGATTT<br>CCCAATATTACAAATCTCTGTCCGTTCGGAGAGGTCTTTAATGCTACCCGATTCGCGTCAGT<br>ATACGCCTGGAACAGAAAGAGAATTTCTAACTGTGTTGCAGATTATAGTGTCCTGTATAATT<br>CTGCGTCTTTTAGCACTTTTAAGTGCTACGGCGTTAGCCCCACTAAGTTGAACGACCTTTGT<br>TTCACTAACGTGTATGCCGACTCATTCGTCATAAGAGGCGACGAAGTTAGACAAATTGCAC<br>CGGGCCAGACGGGAAAGATTGCGGACTACAACTATAAATTGCCTGACGACTTTACAGGATG<br>TGTCATCGCCTGGAATAGTAATAACCTTGACTCCAAAGTCGGTGGCAATTACAATTACTTGT<br>ACCGGCTGTTCAGGAAGTCTAATCTCAAACCTTTTGAGCGAGATATCAGCACGGAAATTTA<br>TCAAGCTGGTAGCACTCCATGTAACGGGGTTGAGGGTTTTAATTGTTATTTTCCATTGCAAT<br>CATATGGATTCCAACCGACTAACGGTGTTGGGTATCAACCATACAGAGTGGTGGTTTTGTCA<br>TTTGAACTTCTGCATGCCCCTGCAACAGTGTGCGGACCGAAGAAGAGTACGAACCTTGTAA<br>AGAACAAGTGCGTCAACTTCAACTTTAATGGTCTGACGGGTACCGGCGTTCTGACGGAATC<br>CAATAAAAAGTTCTTGCCCTTTCAGCAGTTCGGGCGAGATATCGCCGACACTACTGATGCG<br>GTGCGAGATCCTCAGACACTTGAGATCCTCGATATTACCCCATGTAGTTTTGGTGGTGTGTC<br>TGTGATTACACCCGGCACCAATACGTCAAATCAGGTCGCAGTCTTGTACCAAGACGTGAAC<br>TGCACCGAAGTTCCTGTAGCCATTCACGCTGATCAATTGACACCGACATGGAGGGTGTACT<br>CCACCGGATCTAACGTGTTCCAGACCCGCGCGGGGTGTCTTATCGGCGCAGAACATGTGAA<br>CAACTCTTACGAATGTGATATTCCTATCGGTGCAGGCATCTGTGCCTCATACCAGACACAAA<br>CGAACTCACCAAGGAGGGCAAGGTCAGTAGCCTCACAAAGCATAATAGCCTATACGATGA<br>GTCTTGGTGCGGAGAACTCTGTGGCGTACTCTAATAACTCTATCGCCATACCGACTAACTTC<br>ACCATTTCTGTTACGACCGAGATCCTCCCAGTTTCCATGACTAAGACAAGTGTGGATTGTAC<br>AATGTACATCTGCGGCGACAGTACTGAGTGCAGTAACCTGCTTCTGCAGTACGGGTCCTTCT<br>GCACACAACTTAACCGGGCGCTGACTGGTATAGCGGTTGAACAAGACAAGAACACTCAAG<br>AGGTCTTCGCACAAGTAAAACAAATATACAAAACACCACCTATTAAAGATTTCGGCGGGTT<br>TAATTTTAGCCAAATCCTTCCAGACCCCAGCAAACCCTCTAAGCGCAGCTTCATTGAGGATC<br>TGCTGTTTAACAAGGTCACCCTGGCAGACGCGGGCTTTATCAAGCAATACGGTGACTGCCT<br>GGGGGATATCGCGGCTCGAGACCTTATATGTGCGCAAAAATTTAATGGACTTACCGTACTT<br>CCTCCATTGCTGACTGACGAGATGATAGCACAGTATACATCTGCACTGCTCGCCGGTACAAT<br>TACATCAGGGTGGACATTTGGGGCGGGAGCTGCGCTCCAGATACCGTTCGCGATGCAGATG<br>GCGTATAGGTTTAATGGAATTGGTGTCACGCAAAACGTTCTCTATGAAAACCAGAAGCTGA<br>TAGCAAATCAGTTCAATTCCGCGATTGGTAAGATACAAGATTCATTGTCTAGTACGGCCTCT<br>GCACTCGGAAAACTCCAAGATGTAGTGAACCAAAACGCCCAAGCCCTGAATACACTCGTAA<br>AACAGCTCTCTAGTAATTTTGGGGCCATTTCCTCCGTATTGAACGACATCTTGAGTCGCTTG<br>GATAAGGTAGAAGCAGAAGTACAAATTGACCGGTTGATCACGGGCAGACTTCAATCACTTC<br>AGACTTATGTTACTCAGCAGCTTATACGAGCTGCAGAAATTCGCGCCTCTGCGAACCTGGCC<br>GCCACTAAAATGTCAGAATGTGTACTGGGACAGAGCAAACGGGTGGATTTCTGCGGAAAG<br>GGCTATCATCTGATGAGTTTTCCCCAGTCTGCGCCTCATGGTGTAGTATTTCTTCATGTCACA<br>TATGTACCAGCCCAAGAAAAAAATTTCACAACGGCGCCCGCGATTTGCCATGACGGTAAGG<br>CGCATTTTCCTCGCGAGGGCGTTTTCGTGTCTAACGGTACTCACTGGTTCGTAACACAGCGA<br>AACTTTTACGAGCCTCAGATAATCACGACGGATAACACATTTGTCTCCGGCAACTGCGATGT<br>GGTCATCGGTATAGTGAACAATACGGTATATGATCCGCTGCAGCCAGAGCTCGACAGTTTC<br>AAGGAGGAGCTTGACAAATACTTTAAGAACCATACCTCCCCAGACGTAGACCTCGGAGACA

	TATCTGGTATCAATGCCTCCGTGGTTAACATACAAAAGGAGATAGATAGACTGAATGAGGT<br>GGCGAAGAATCTGAATGAGTCTCTCATAGATCTGCAGGAACTCGGTAAATATGAACAATAC<br>ATCAAGTGGCCTTGGTACATCTGGCTGGGGTTCATAGCGGGCCTGATCGCGATCGTGATGG<br>TAACTATAATGTTGTGTTGCATGACCTCCTGCTGCTCATGCCTTAAAGGTTGTTGTTCTTGCG<br>GGAGCTGCTGCAAGTTCGATGAGGATGATTCAGAACCCGTCTTGAAGGGCGTAAAACTTCA<br>CTATACGTAA	
Primers used to sequence full length SARS-CoV-2-S protein	EF1aFor	GGATCTTGGTTCATTCTCAAG
	SPseqF1	GTACCTGCAACCGAGAAC
	SPseqF2	GGCGTTCTGACGGAATC
	SPseqF3	GCAATACGGTGACTGCC
	SPseqF4	CGTGTCTAACGGTACTCAC
	SPseqR1	GTTCTCGGTTGCAGGTAC
	IRESrev	CATATAGACAAACGCACACC
