# Supplementary material for: Sensitive detection of total anti-Spike antibodies and isotype switching in asymptomatic and symptomatic individuals with COVID-19
Source: Cell Rep Med. 2021 Jan 16;2(2):100193. doi: 10.1016/j.xcrm.2021.100193 (PMC7816583; doi:10.1016/j.xcrm.2021.100193)
Supplement: Document S2. Article plus supplemental information [file mmc2.pdf]

Cell Reports Medicine

Article

Sensitive detection of total anti-Spike antibodies and isotype switching in asymptomatic and symptomatic individuals with COVID-19

Graphical Abstract

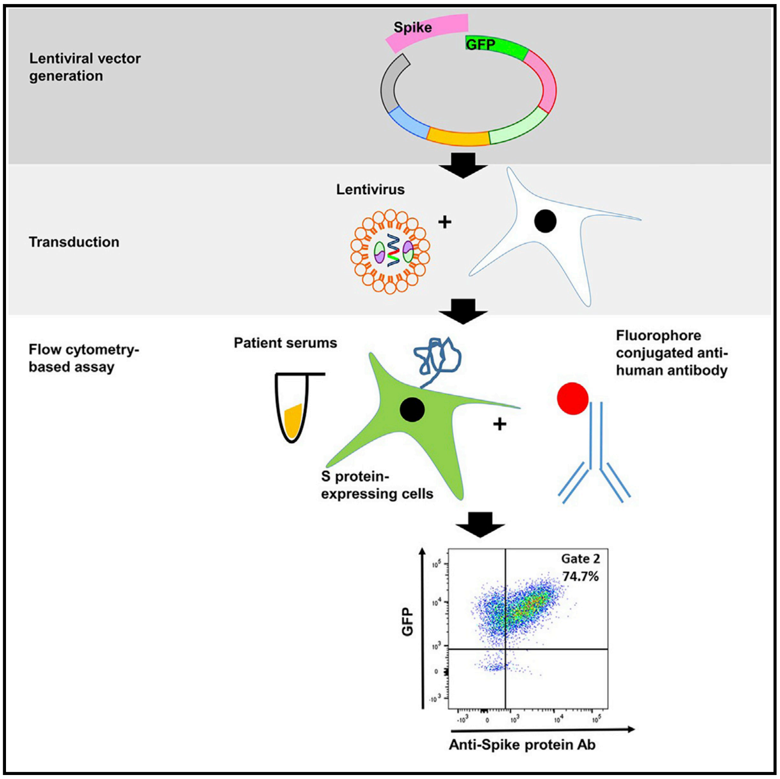

Highlights

- Flow cytometry assay detects specific antibodies in symptomatic individuals with COVID-19
- Asymptomatic individuals with COVID-19 also develop specific antibodies
- IgG1 is the dominant IgG subclass in symptomatic and asymptomatic individuals
- The assay is highly sensitive and detects 97% of asymptomatic infections

Authors

Yun Shan Goh, Jean-Marc Chavatte, Alicia Lim Jieling, ..., Lisa F.P. Ng, Raymond Tzer Pin Lin, Laurent Renia

Correspondence

renia_laurent@immunol.a-star.edu.sg

In Brief

Using a flow cytometry-based assay, Goh et al. find specific antibodies in plasma samples from symptomatic individuals with COVID-19. IgG1 is the most dominant IgG subclass. Despite lower antibody levels, the assay detects 97% of asymptomatic infections, suggesting that the assay detects asymptomatic infections, which may otherwise remain undetected.

Goh et al., 2021, Cell Reports Medicine *2*, 100193
February 16, 2021 © 2021 The Author(s).
https://doi.org/10.1016/j.xcrm.2021.100193

CellPress

Cell Reports Medicine

CellPress OPEN ACCESS

Article

Sensitive detection of total anti-Spike antibodies and isotype switching in asymptomatic and symptomatic individuals with COVID-19

Yun Shan Goh,[1,2] Jean-Marc Chavatte,[3] Alicia Lim Jieling,[3] Bernett Lee,[2] Pei Xiang Hor,[1,2] Siti Naqiah Amrun,[1,2] Cheryl Yi-Pin Lee,[1,2] Rhonda Sin-Ling Chee,[1,2] Bei Wang,[2] Chia Yin Lee,[2] Eve Zhi Xian Ngoh,[2] Cheng-I Wang,[2] Barnaby Edward Young,[3,4,5] Paul A. Tambyah,[3,6] Shirin Kalimuddin,[7,8] Surinder Pada,[9] Seow-Yen Tan,[10] Louisa Jin Sun,[11] Mark I-Cheng Chen,[3,12] Yee-Sin Leo,[3,4,5,13] David C. Lye,[3,4,5,13] Lisa F.P. Ng,[1,2,14,15,16,17] Raymond Tzer Pin Lin,[3,13,18] and Laurent Renia[1,2,17,18,19,*]

[1]Infectious Diseases Laboratories (ID Labs), Agency for Science, Technology and Research (A*STAR), Immunos, Biopolis, Singapore 138648, Singapore

[2]Singapore Immunology Network, Agency for Science, Technology and Research (A*STAR), Immunos, Biopolis, Singapore 138648, Singapore

[3]National Centre for Infectious Diseases, 16 Jalan Tan Tock Seng, Singapore 308442, Singapore

[4]Department of Infectious Diseases, Tan Tock Seng Hospital, 11 Jalan Tan Tock Seng, Singapore 308433, Singapore

[5]Lee Kong Chian School of Medicine, Nanyang Technological University, 11 Mandalay Road, Singapore 308232, Singapore

[6]Department of Medicine, National University Hospital, 5 Lower Kent Ridge Road, Singapore 119074, Singapore

[7]Department of Infectious Diseases, Singapore General Hospital, 31 Third Hospital Ave, Singapore 168753, Singapore

[8]Emerging Infectious Disease Program, Duke-NUS Medical School, 8 College Road, Singapore 169857, Singapore

[9]Division of Infectious Diseases, Ng Teng Fong Hospital, 1 Jurong East Street 21, Singapore 609606, Singapore

[10]Department of Infectious Diseases, Changi General Hospital, 2 Simei Street 3, Singapore 529889, Singapore

[11]Alexandra Hospital, 378 Alexandra Road, Singapore 159964, Singapore

[12]Saw Swee Hock School of Public Health, National University of Singapore and National University Health System, 12 Science Drive 2, Singapore 117549, Singapore

[13]Yong Loo Lin School of Medicine, National University of Singapore and National University Health System, 10 Medical Drive, Singapore 117597, Singapore

[14]Department of Biochemistry, Yong Loo Lin School of Medicine, National University of Singapore, Singapore 117596, Singapore

[15]National Institute of Health Research, Health Protection Research Unit in Emerging and Zoonotic Infections, University of Liverpool, Liverpool, UK

[16]Institute of Infection, Veterinary and Ecological Sciences, University of Liverpool, Liverpool, UK

[17]Department of Microbiology and Immunology, Yong Loo Lin School of Medicine, National University of Singapore, Singapore 117597, Singapore

[18]Senior author

[19]Lead contact

*Correspondence: renia_laurent@immunol.a-star.edu.sg

https://doi.org/10.1016/j.xcrm.2021.100193

SUMMARY

Early detection of infection is crucial to limit the spread of coronavirus disease 2019 (COVID-19). Here we develop a flow cytometry-based assay to detect severe acute respiratory syndrome coronavirus 2 (SARS-CoV-2) spike (S) protein antibodies in individuals with COVID-19. The assay detects specific immunoglobulin M (IgM), IgA, and IgG in individuals with COVID-19 and also acquisition of all IgG subclasses, with IgG1 being the most dominant. The antibody response is significantly higher at a later stage of infection. Furthermore, asymptomatic individuals with COVID-19 also develop specific IgM, IgA, and IgG, with IgG1 being the most dominant subclass. Although the antibody levels are lower in asymptomatic infection, the assay is highly sensitive and detects 97% of asymptomatic infections. These findings demonstrate that the assay can be used for serological analysis of symptomatic and asymptomatic infections, which may otherwise remain undetected.

INTRODUCTION

Coronavirus disease 2019 (COVID-19) is a major health issue affecting 216 countries, with 34 million confirmed cases of human infection and more than 1 million fatalities so far.[1] By April 2020, a mere 4 months after the first case was reported, COVID-19 has developed into a pandemic that has led to partial or total confinement of more than half of the world's population.

© 2021 The Author(s). This is an open access article under the CC BY license (http://creativecommons.org/licenses/by/4.0/).

This unprecedented crisis has resulted in overwhelmed healthcare systems and major socio-economic disruption.

One of the key public health strategies to control the spread of COVID-19 is early detection of infected individuals to allow early blocking of transmission of severe acute respiratory syndrome coronavirus 2 (SARS-CoV-2).[2–4] Although quantitative reverse-transcriptase PCR (qRT-PCR) remains the gold standard for COVID-19 diagnosis,[5,6], it can be time-consuming, and insufficient viral genetic material at the point of detection because of inefficient sample preparation or low viral load in an individual can lead to false negative diagnosis. Although prolonged virus shedding has been detected in some people,[7] SARS-CoV-2 viral RNA becomes almost undetectable in most individuals by 14 days post-illness onset (pio).[8–10]

Serological assays that detect specific antibodies against SARS-CoV-2[10,11] have increasingly been utilized to complement PCR-based assays, especially for symptomatic infections with a low viral load. As of August 17, 2020, the US Food and Drug Administration (FDA) approved use of 37 serological assays[12] mainly targeting two immunogenic proteins: the spike (S) protein,[1] the most exposed viral protein, and the nucleocapsid (N) protein,[2] which is expressed abundantly during infection.[13–15] The S protein is responsible for binding and entry of the virus into the host cell via the cellular receptor angiotensin-converting enzyme 2 (ACE2).[16] Many S protein-based serological assays are ELISAs, which are based on linear peptides, to detect specific antibodies to the S1 subunit, S2 subunit, or receptor binding domain (RBD),[14,17–19] which may not capture the full repertoire of antibodies, such as antibodies binding to various domains and conformational epitopes of the S protein.

Here we developed a sensitive and time-efficient flow cytometry-based assay that detects antibodies against the full-length SARS-CoV-2 S protein. Using lentivirus-transduced cells that stably express the full-length S protein on the cell surface, we aimed to examine a wider repertoire of antibodies against the S protein over the course of infection. We also studied the immunoglobulin G (IgG) subclass response to the S protein to understand the involvement of different IgG subclasses in immunity against COVID-19.

RESULTS

Profiles of specific antibodies against full-length S protein over the course of infection

To characterize the antibody profile of individuals with COVID-19, we developed a flow cytometry assay based on the full-length SARS-CoV-2 S protein (SFB, S protein flow-based assay), which allows detection of a wider repertoire of antibodies, such as antibodies binding to various domains and conformational epitopes of the S protein. To this end, we transduced HEK293T cells with lentiviral particles to stably express the full-length S protein on the cell surface. We verified expression of the S protein on the cell surface by examining binding of ACE2-huFc (ACE2 protein tagged with a human Fc) and S protein RBD-specific monoclonal antibody clone 5A6[20] to S protein-expressing cells (Figure S1).

Using the SFB assay, we examined the antibody response to the full-length S protein in symptomatic individuals with COVID-19 (n = 81; Table S1) over the course of infection at time points with a median of 5 days, 10 days, and 23 days pio. Various control groups (Table S1) were assessed in parallel: (1) recovered SARS individuals (n = 20), (2) healthy controls (n = 22), and (3) individuals with seasonal human CoV (n = 20).[3] For the assay, plasma samples were screened for specific S protein antibodies by first incubating lentivirus-transduced cells with diluted plasma samples, followed by a secondary incubation with fluorophore-conjugated anti-human antibody to detect antibody binding. Specific IgM against the S protein was detected in individuals with COVID-19, with the response being higher at a median of 10 and 23 days pio than a median of 5 days pio (Figure 1A). A total of 32% of the individuals had a positive IgM response as early as a median of 5 days pio (Figure 1D). By a median of 10 days pio, 63% of the individuals acquired IgM, whereas 92% acquired IgM at a median of 23 days pio. Notably, specific IgM was not detected in all control groups, highlighting the specificity of the assay (Figures 1A and 1D). Similar to IgM, a specific IgA response was detected as early as a median of 5 days pio, with the highest response at a median of 23 days pio (Figure 1B). A specific IgA response was detected in 35% of individuals at a median of 5 days pio, 66% at a median of 10 days pio, and 98% at a median of 23 days pio (Figure 1E). The specific IgG response was also highest at a median of 23 days pio (Figure 1C). IgG seroconversion was detected in 47% of individuals at a median of 5 days pio and 86% at a median of 10 days pio (Figure 1F). By a median of 23 days pio, 100% seroconversion was achieved. Four recovered SARS individuals also demonstrated IgG cross-reactivity against the SARS-CoV-2 S protein (Figures 1C and 1F).

IgG1 is the dominant IgG subclass specific against the S protein

We then studied the specific S protein IgG subclass profiles of individuals with COVID-19. All four IgG subclasses were detected, with responses being highest at a median of 23 days pio (Figures 2A–2D). There was a dominance of IgG1 responses, followed by IgG3, IgG2, and IgG4 (Figures 2E–2G). Seroconversion of IgG subclasses was lower at a median of 5 and 10 days (Figures 2H and 2I). By a median of 23 days pio, all individuals had a positive IgG1 response, whereas 74%, 94%, and 67% had a positive IgG2, IgG3, and IgG4 response, respectively (Figure 2J). This demonstrated isotype switching of all IgG subclasses against the S protein over time in individuals with COVID-19, with IgG1 being the most dominant IgG subclass.

The SFB assay is specific and sensitive

We next assessed the utility of the SFB assay for serological diagnosis of SARS-CoV-2. Using the control groups (recovered SARS, n = 20; healthy controls, n = 22; seasonal human CoV, n = 20), the specificity of the SFB assay was 100%, 98%, and 93% for IgM, IgA, and IgG detection, respectively (Table 1), for all three time points. No cross-reactivity was observed for IgM detection. Cross-reactivity was observed in 1 of 22 healthy controls for IgA detection. For IgG, cross-reactivity was observed in 4 of 20 recovered SARS individuals but not in healthy controls or individuals with seasonal human CoV. The specificity of the SFB assay was 97%, 98%, 98%, and 98% for IgG1, IgG2, IgG3, and

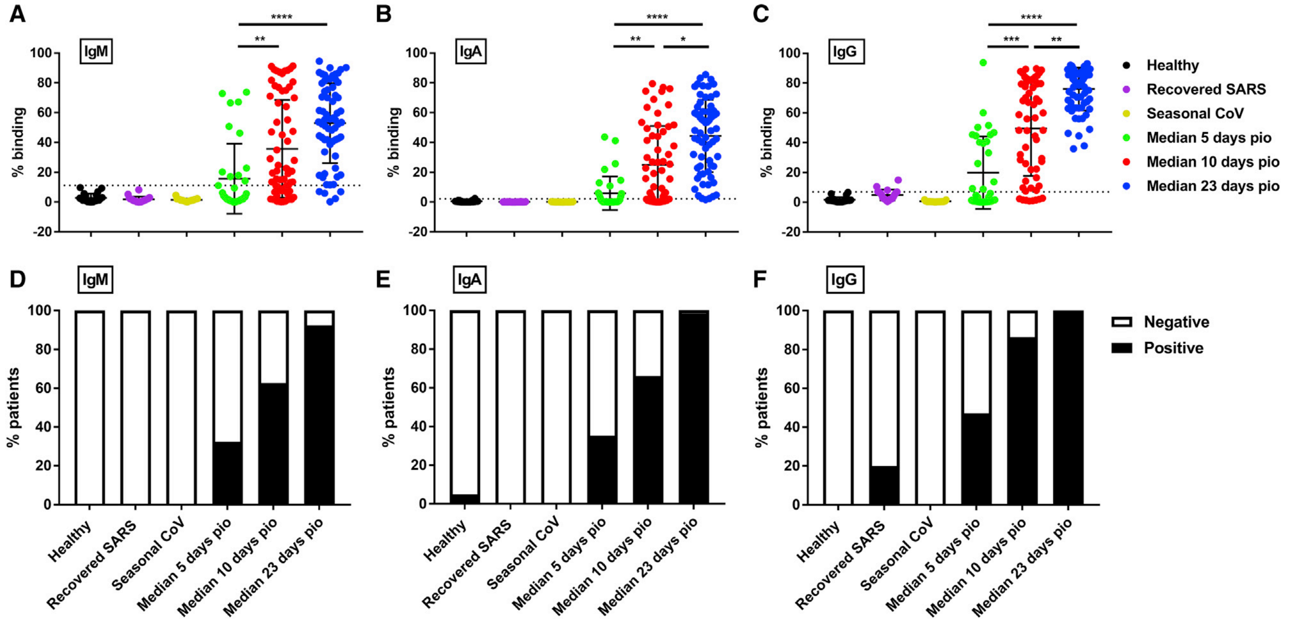

Figure 1. Specific antibodies against full-length S protein

Plasma samples were collected from individuals with COVID-19 (n = 81) at time points with a median of 5 days post-illness onset (pio, n = 34), 10 days pio (n = 59), and 23 days pio (n = 66).

(A–C) Samples were screened at 1:100 dilution for specific (A) IgM, (B) IgA, and (C) IgG against full-length SARS-CoV2 S protein expressed on the surface of HEK293T cells. Control samples included plasma samples from recovered SARS individuals (recovered SARS, n = 20), plasma samples from healthy donors (healthy, n = 22), and sera from seasonal human CoV-infected individuals (seasonal CoV, n = 20).

(D–F) The proportions of individuals having a positive (D) IgM, (E) IgA, and (F) IgG response were analyzed.

Data are shown as mean ± SD of two independent experiments, with dotted lines indicating mean + 3 SDs of healthy donors. An isotype response was defined as positive by SFB assay when binding was more than mean + 3 SDs of the healthy controls. Statistical analysis was carried out using Kruskal-Wallis tests followed by post hoc Dunn's multiple comparisons tests. The p values for comparisons between the different time points are shown; *$p \leq 0.05$, **$p \leq 0.01$, ***$p \leq 0.001$, ****$p \leq 0.0001$.

IgG4, respectively, for all three time points. For IgG1, we observed cross-reactivity in 1 of 20 recovered SARS individuals and 1 of 22 healthy controls. For IgG2, IgG3, and IgG4, cross-reactivity was detected in 1 of 22 healthy controls.

Using the cohort of 81 symptomatic individuals with COVID-19 (Table 1), the sensitivity of the SFB assay for IgM detection (32%, 63%, and 92% at a time point of a median of 5 days pio, 10 days pio, and 23 days pio, respectively) and IgA detection (35%, 66%, and 98% at a time point of a median of 5 days pio, 10 days pio, and 23 days pio, respectively) was comparable. The SFB assay was more sensitive for IgG detection: 47%, 86%, and 100% at a median of 5 days, 10 days, and 23 days pio, respectively (Table 1). As expected, at a median of 5 and 10 days pio, when antibody responses were lower, the sensitivity of the SFB assay for IgG1, IgG2, IgG3, and IgG4 detection was also lower. At a later time point of a median of 23 days pio, the SFB assay was more sensitive for IgG1 and IgG3 detection (100% and 94%, respectively) but less sensitive for IgG2 and IgG4 detection (74% and 63%, respectively).

The SFB assay can detect pre/asymptomatic infection

Having established the SFB assay for serological analysis of symptomatic infection, we proceeded to further examine the sensitivity of the assay and evaluate the applicability of the assay to serological diagnosis of samples with limited clinical information. The Singapore National Public Health Laboratory (NPHL) received samples collected from convalescent individuals, individuals with suspected infection, and general populations for sero-prevalence studies. A total of 109 samples, grouped by PCR status and symptom status (Table S1), were screened: (1) PCR-positive and symptomatic, n = 16; (2) PCR-positive and pre/asymptomatic, n = 34; (3) PCR-positive and unknown symptom status, n = 11; (4) PCR-negative and unknown symptom status, n = 13; (5) PCR-negative/PCR not done and no symptoms, n = 20; and (6) PCR status unknown and unknown symptom status, n = 15.

In agreement with our findings with the earlier cohort of 81 symptomatic individuals with COVID-19, the SFB assay detected IgM (Figure 3A), IgA (Figure 3B), IgG (Figure 3C) and IgG subclasses (Figures 3D–3G) against the S protein in PCR-positive and symptomatic infections. More importantly, the assay also detected specific IgM and IgA and IgG subclasses against the S protein in PCR-positive and pre/asymptomatic infections, with IgG1 being the dominant IgG subclass. The antibody response in this group was significantly lower than that observed with samples from PCR-positive and symptomatic infections for all isotypes. Similarly, for the rest of the four groups, all isotypes responses were lower than that observed with samples from PCR-positive and symptomatic infections. IgG1 was also the

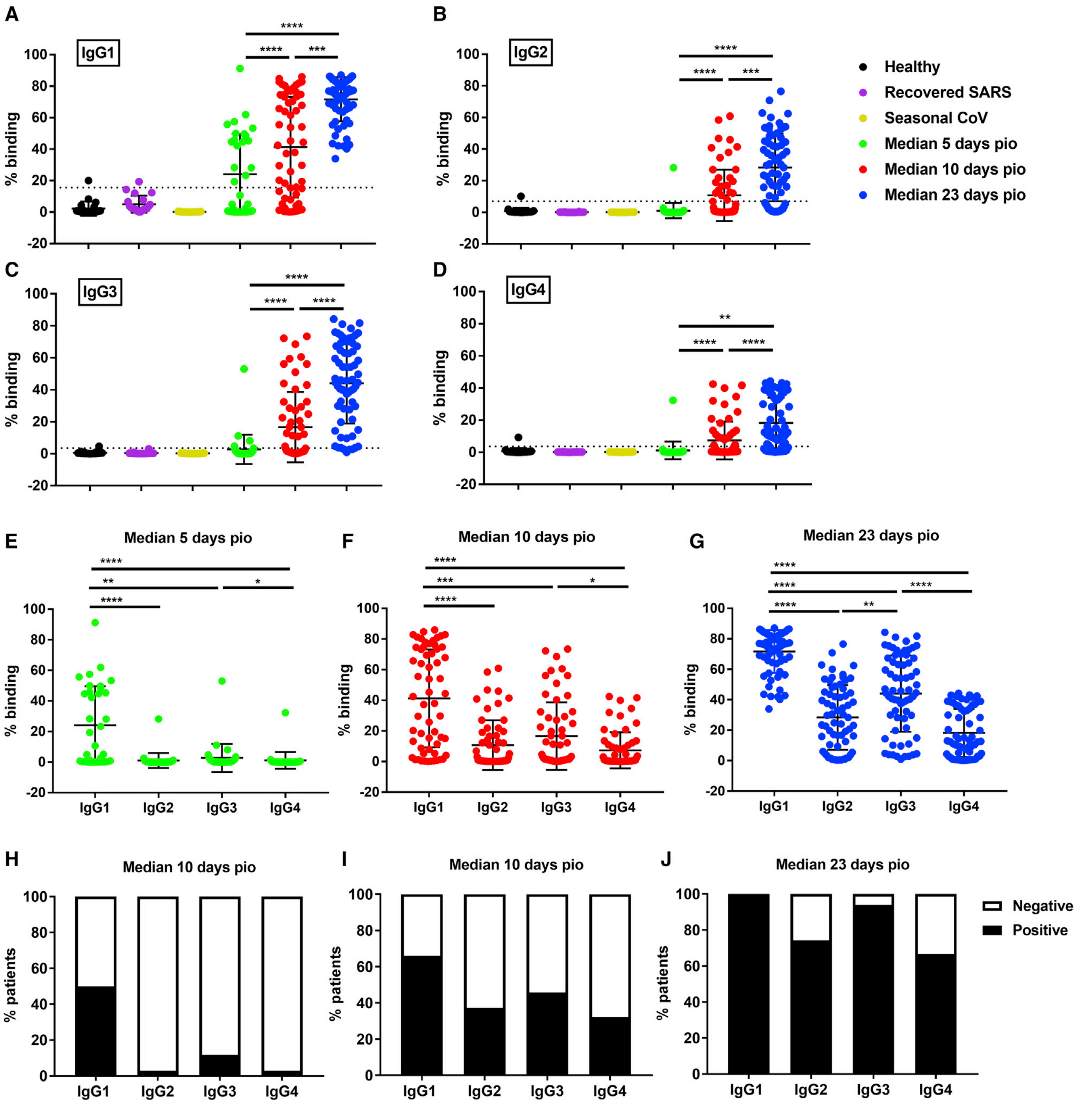

Figure 2. Specific IgG subclasses against full-length S protein

(A–D) Plasma samples collected from 81 individuals with COVID-19 at time points with a median of 5 days pio (n = 34), 10 days pio (n = 59), and 23 days pio (n = 66) were further screened for IgG subclasses, (A) IgG1, (B) IgG2, (C) IgG3, and (D) IgG4.

(E–G) The four IgG subclass responses at a median of (E) 5 days pio, (F) 10 days pio, and (G) 23 days pio were plotted.

(H–J) The proportions of individuals having a positive response at time points of (H) a median of 5 days, (I) a median of 10 days, and (J) a median of 23 days pio were analyzed.

Data are shown as mean ± SD of two independent experiments, with dotted lines indicating mean + 3 SDs of healthy donors. An isotype response was defined as positive by SFB assay when the binding was more than mean + 3 SDs of the healthy controls. Statistical analysis was carried out using Kruskal-Wallis tests followed by post hoc Dunn's multiple comparisons tests. For (A)–(D), only p values for comparisons between the different time points are shown; *p $\leq$ 0.05, **p $\leq$ 0.01, ***p $\leq$ 0.001, ****p $\leq$ 0.0001.

Table 1. Receiver operating characteristic (ROC) profiles of specific S protein antibodies

	Isotype	Threshold[a] (%)	Sensitivity (%)	Specificity (%)	Area under the ROC Curve (AUC)
Median 5 days pio	IgM	11.28	32.35	100.00	0.714
	IgA	2.16	35.29	98.36	0.747
	IgG	6.93	47.06	93.44	0.714
	IgG1	15.57	50.00	96.72	0.735
	IgG2	7.04	2.94	98.36	0.434
	IgG3	3.50	11.76	98.36	0.528
	IgG4	6.38	2.94	98.36	0.399
Median 10 days pio	IgM	11.28	62.71	100.00	0.886
	IgA	2.16	66.10	98.36	0.931
	IgG	6.93	86.44	93.44	0.949
	IgG1	15.57	66.10	96.72	0.898
	IgG2	7.04	37.29	98.36	0.914
	IgG3	3.50	45.76	98.36	0.903
	IgG4	6.38	32.20	98.36	0.909
Median 23 days pio	IgM	11.28	92.42	100.00	0.982
	IgA	2.16	98.49	98.36	0.999
	IgG	6.93	100.00	93.44	1.000
	IgG1	15.57	100.00	96.72	1.000
	IgG2	7.04	74.24	98.36	0.995
	IgG3	3.50	93.94	98.36	0.997
	IgG4	6.38	62.71	98.36	0.988

[a]Threshold is defined as mean + 3 SDs of healthy controls (n = 22).

dominant IgG subclass, whereas the levels of IgG2 and IgG4 were negligible. Interestingly, for all groups, we observed a significantly higher IgG1 response compared with the total IgG response (Figures 4A–4F). This suggests that IgG1 detection with the SFB assay might provide greater sensitivity.

Comparison of the SFB assay with commercially available serological assays

All samples received at the NPHL were first assessed by two commercially available serological assays: (1) the GenScript cPass S Protein RBD Neutralization Antibody Detection Kit because of its ability to detect neutralizing antibodies targeting the RBD of the S protein and (2) the Wondfo SARS-CoV-2 antibody rapid diagnostic test (RDT) with undisclosed antigen specificity because of the rapid test format and ease of application to high-throughput screening (HTS). With the exception of samples collected from PCR-positive and symptomatic individuals (which tested positive with the two commercial antibody assays), most of the remaining 93 samples yielded borderline or discrepant results with the two commercial assays and were selected for analysis by the SFB assay. Because both commercially available serological assays do not differentiate between individual isotype positivity, the sample is defined as positive by the SFB assay when any one of the seven isotypes (IgM, IgA, IgG, IgG1, IgG2, IgG3, or IgG4) is positive.

In comparison, we found the SFB assay to be highly sensitive. With PCR-positive and symptomatic infections, the SFB assay was comparable with the two commercially available assays, detecting 100% of the infections (Figure 4G). More importantly, the SFB assay was able to detect 97% of PCR-positive and pre/asymptomatic infections compared with 32% and 35% with the cPass and RDT assays, respectively. For PCR-positive samples and samples with unknown symptom status, the SFB assay detected 100% of samples compared with 18% with the cPass assay. Similarly, for the rest of the three groups, the SFB assay was more sensitive than the cPass and RDT assays (Figure 4G), able to detect 62% of the PCR-negative samples and samples with unknown symptom status, 95% of the PCR-negative/PCR not done and no symptom samples, and 80% of samples with unknown PCR status and unknown symptom status. These findings demonstrated the high sensitivity of the SFB assay, suggesting that the assay could be an instrumental tool to detect asymptomatic infection.

DISCUSSION

The current strategy for controlling the COVID-19 pandemic requires confining the world's population, which is not sustainable in the long term. Gradual easing of control measures will require active surveillance of the population to ensure early detection of new infections, contact tracing and quarantine, and continued social distancing measures to block transmission. Serological assays are instrumental in confirming symptomatic infections and detecting individuals who are pre-symptomatic or asymptomatic or have recovered.

We previously developed an ELISA-based serological assay based on the four immunodominant IgG linear epitopes on the S and N proteins.[21] To capture a wider repertoire of antibodies

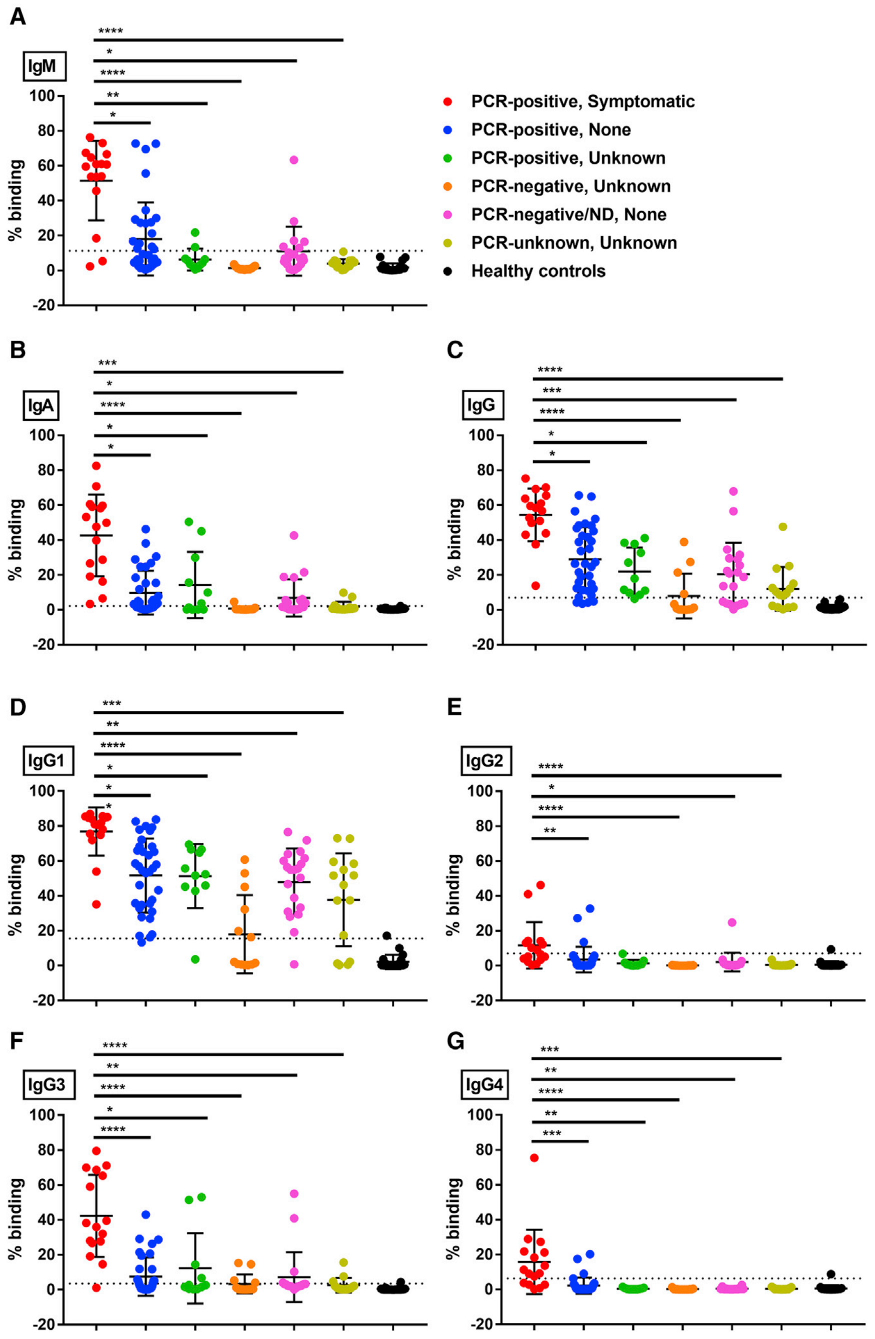

Figure 3. S protein-specific antibody profile in pre/asymptomatic infections

(A–G) Plasma samples were collected from individuals with suspected COVID-19 infection or randomly from the general population under the Singapore Infectious Disease Act. Samples were screened at 1:100 dilution for specific (A) IgM, (B) IgA, (C) IgG, (D) IgG1, (E) IgG2, (F) IgG3, and (G) IgG4 against full-length SARS-CoV2 S protein expressed on the surface of HEK293T cells. A total of 109 samples were analyzed and included sera from PCR-positive and symptomatic individuals (PCR-positive, symptomatic; n = 16), sera from PCR-positive and no-symptom individuals (PCR-positive, none; n = 34), sera from PCR-positive individuals and individuals with unknown symptom status individuals (PCR-positive, unknown; n = 11), sera from PCR-negative individuals and individuals with unknown symptom status (PCR-negative, unknown; n = 13), sera from PCR-negative individuals/PCR not done and individuals with no symptoms (PCR-negative/not done [ND], none; n = 20), and sera from individuals with unknown PCR status and unknown symptom status (PCR unknown, unknown; n = 15). Statistical analysis was carried out using Kruskal-Wallis tests followed by post hoc Dunn's multiple comparisons tests. The p values for comparisons of PCR-positive, symptomatic, and other groups are shown; $^{*}p \leq 0.05$, $^{**}p \leq 0.01$, $^{***}p \leq 0.001$, $^{****}p \leq 0.0001$.

against the S protein, in this study we developed a flow cytometry assay based on the full-length S protein. The assay is more time efficient; results are available within 2 h. The assay is also well suited to detect anti-S protein antibodies in symptomatic and asymptomatic individuals. Symptomatic individuals with COVID-19 acquired specific IgM, IgA and IgG over the course of infection, with all individuals having a detectable antibody response at a later stage of infection (a median of 23 days pio) (Figure 1).

One defining feature of the assay is its ability to detect specific IgG subclasses against the S protein. In our cohort, we found that all IgG subclasses were acquired by individuals with COVID-19, with IgG1 being the most dominant. Similar to total IgG, the IgG subclass response was significantly greater at a later stage of infection. This is in agreement with a study from Ni et al.,[22] who also found a predominant IgG1 response against the RBD of the S protein and also the N protein. Inclusion of IgG subclasses in serological detection is important and bridges knowledge gaps in understanding protective immunity against COVID-19 and the likelihood of protection from re-infection. IgG1 and IgG3 induction, typically indicative of a T helper type 1 (TH1) response,[23] is a pro-inflammatory response particularly important in protective immunity against viruses. IgG1 and IgG3 possess higher neutralization capabilities against many different viruses.[24–26]

With increasing reports of asymptomatic individuals having a similar transmission capability as symptomatic individuals,[27,28] it is crucial to also stem asymptomatic transmission for effective COVID-19 control. We found that the SFB assay was able to detect 97% of pre/asymptomatic infections. The SFB assay could be a more effective tool for detecting COVID-19 infection and might be more informative for determining exposure. Although the sensitivity of the cPass assay[29] and the RDT

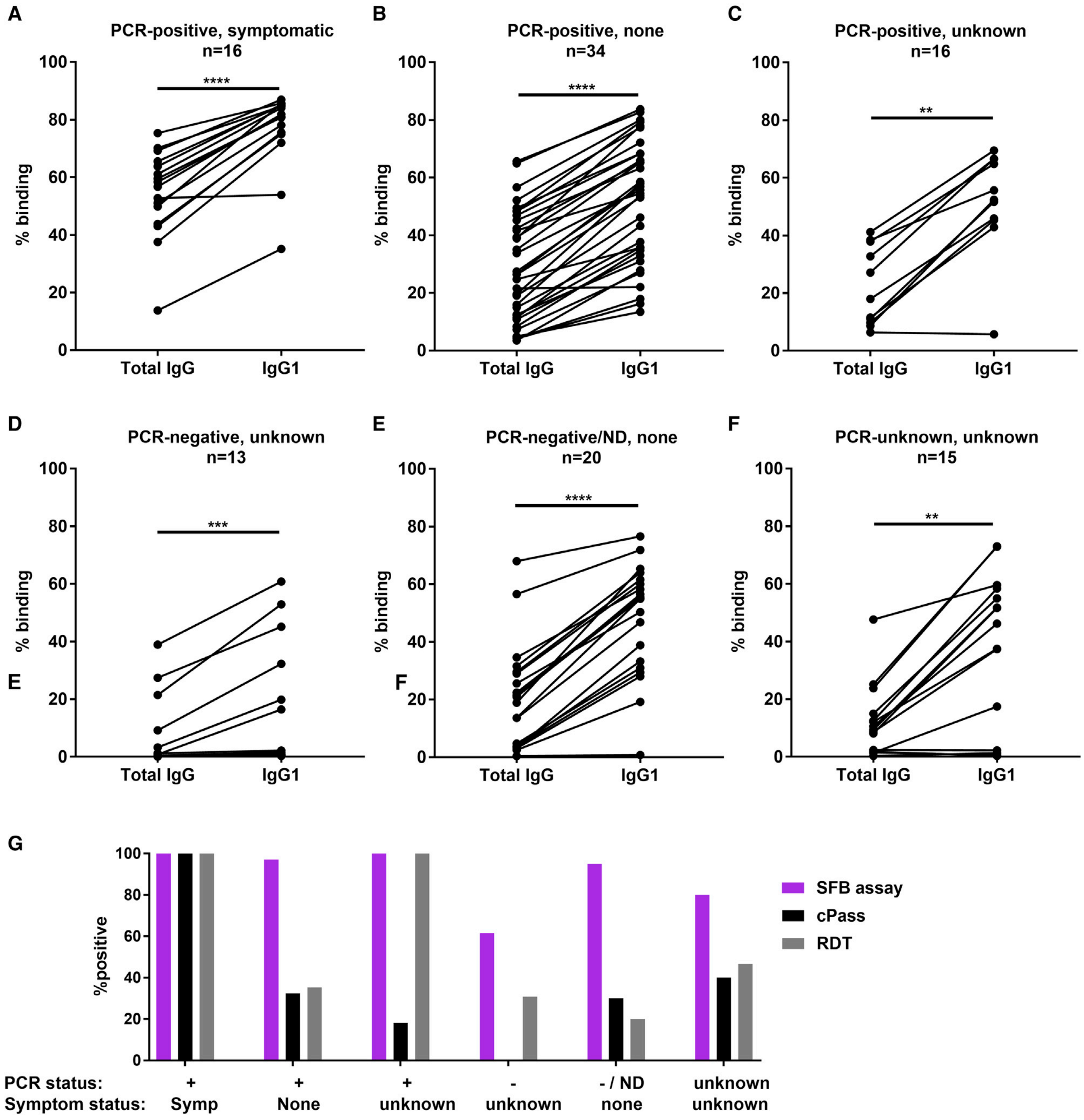

Figure 4. Comparison of total IgG and IgG1 responses and comparison of the SFB assay and other serological assays

(A–F) The total IgG response was compared with the IgG1 responses for all 109 samples from the NPHL: (A) PCR-positive and symptomatic infections (PCR-positive, symptomatic; n = 16), (B) PCR-positive and no-symptom infections (PCR-positive, none; n = 34), (C) PCR-positive and unknown symptom status infections (PCR-positive, unknown; n = 11), (D) PCR-negative and unknown symptom status infections (PCR-negative, unknown; n = 13), (E) PCR-negative/PCR not done and no-symptom infections (PCR-negative/ND, none; n = 20), and (F) PCR unknown and unknown symptom status infections (PCR unknown, unknown; n = 15). Statistical analysis was carried out using Wilcoxon matched-pairs signed-rank test. The p values for comparisons between the total IgG and IgG1 responses are shown; *p $\leq$ 0.05, **p $\leq$ 0.01, ***p $\leq$ 0.001, ****p $\leq$ 0.0001. Data are shown as mean ± SD of two independent experiments, with dotted lines indicating mean + 3 SDs of healthy donors.

(G) Proportion of samples that were positive by serological tests: SFB assay, cPass, and RDT. An isotype response was defined as positive by SFB assay when the binding was more than mean + 3 SDs of the healthy controls. Samples that had a positive response for any one of the seven isotypes (IgM, IgA, IgG, IgG1, IgG2, IgG3, and/or IgG4) were defined as positive by the SFB assay.

Data are shown as mean SD of two independent experiments, with dotted lines indicating mean + 3 SDs of healthy donors. +, positive; −, negative; symp, symptomatic; none, no symptoms.

assay[30] has been reported to be 94% and 86%, respectively, we found that the SFB assay was more sensitive and able to detect specific antibodies in cases where discrepant or borderline results were achieved with the commercial cPass and RDT assays. The antibody response was significantly lower in the PCR-positive and pre/asymptomatic individuals than in symptomatic people, which is in agreement with a recent study.[31] Despite the lower antibody levels, the assay was able to detect 97% of these infections, whereas the cPass and RDT assays did not yield clear serological outcomes. This showed that more sensitive assays, such as the SFB assay, are needed to detect pre/asymptomatic infections where the antibody response is weaker and especially for determining exposure in the population, The higher sensitivity of the SFB assay over the cPass assay could be attributed to the target of the assay; the SFB assay is based on the full-length S protein, which allows capture of antibodies against various domains and conformational epitopes, whereas the cPass assay was designed to detect specifically neutralizing antibodies against the RBD of the S protein that block the interaction between the RBD domain and ACE2, its receptor on the host cell. The target of the RDT has not been disclosed. It is possible that it is also based on a particular domain of the S protein, capturing a smaller repertoire of S protein antibodies. It is also possible that the target is a different protein, such as the N protein, and thus recognizes a different set of antibodies with different kinetics in antibody induction. IgG1 subclass detection by the SFB assay was key to ascertain exposure to the virus and provide better sensitivity than testing IgG alone; a significantly greater IgG1 response compared with total IgG was observed in all groups (Figure 4). It is possible that the secondary anti-human IgG antibodies might not bind to all IgG subclasses with similar efficiency. More importantly, the greater IgG1 response (over total IgG) could be due to a prozone effect, where high levels of antibodies result in lack of antigen binding.[32–35] Although there is likely a decrease in IgG levels over time,[36,37] it is also possible that the lack of IgG binding could be, in part, due to a prozone effect. We selected eight samples from the NPHL cohort, where the IgG1 response is greater than the IgG response, and performed the SFB assay with serially diluted samples. We observed a higher IgG response at a higher dilution factor of 300, whereas the IgG1 response was higher at a lower dilution factor of 100 (Figure S2), suggesting that the greater IgG1 response (over IgG) could, in part, be due to a prozone effect. Hence, detection of a subset of total IgG, IgG1 antibodies, might be better than the whole repertoire of total IgG.

Serological assays are complementary to PCR assays for COVID-19 diagnosis. As a preliminary evaluation of the SFB assay for ongoing sero-epidemiological studies, we tested potentially exposed individuals who were PCR negative from our NPHL cohort. The SFB assay detected 8 of 13 (62%) of such samples that were borderline positive or discrepant with the cPass and RDT assays, indicating that a fraction of them have been infected/exposed to the virus. Most of Singapore's COVID-19 cases have been among the migrant worker population[38] living in dormitories where social distancing is difficult. Samples were collected from dormitory residents with no symptoms and a PCR-negative/PCR not done status to assess transmission in dormitories. The SFB assay detected 19 of 20 (95%) such samples that were borderline or discrepant by the cPass and RDT assays. These findings also further demonstrated the high sensitivity of the SFB assay. It is worth noting that, while preparing for this publication, another similar study was published where the authors used transiently transfected cells to express full-length S protein on the cell surface to develop a flow cytometry-based assay.[39] The authors also found high sensitivity with the S protein flow cytometry-based assay, which detected infections in asymptomatic individuals whereas other ELISA-based assays did not.

Our findings demonstrated that the SFB assay could be used for serological confirmation of symptomatic infection. The SFB assay could also be used, in combination with other serological assays, to detect asymptomatic infection and assess sero-prevalence in the community. This would be an instrumental tool for sero-surveillance and provide crucial insight into the extent of undetected and undiagnosed COVID-19 cases in the community. In addition, the SFB assay could also be used to examine the antibody response in previously infected individuals long after they have recovered to have a better understanding of the persistence of antibody-mediated protection against COVID-19. The high sensitivity of the SFB assay is also particularly useful in clinical investigation of suspected infection and epidemiological links within clusters, which might yield borderline/discrepant results or even be missed by less sensitive serological assays. It aids in contact tracing efforts to limit the extent of community spread. This is especially pertinent at a time when governments around the world are looking to gradually reopen the economy. This would greatly help to form better public policy decisions to manage and limit COVID-19 infection.

Limitations of study

Similar to all serological assays, one main limitation of the SFB assay is the risk of false positive diagnosis. Although high sensitivity is needed to detect asymptomatic infections, it is also important to have high specificity. Because the SFB assay consists of seven tests (IgM, IgA, IgG, and four IgG subclasses), it allows internal validation. For 97 of 109 samples tested, a positive response was detected for two or more isotypes. 12 of 109 samples tested had a positive response for only one isotype, where 6 of 12 were also found to be positive by the cPass or RDT assay, and another 2 of 12, although negative by cPass and RDT assay, were PCR positive. This showed that borderline positive results should be interpreted with caution. One other limitation of the SFB assay is the difficulty to apply the assay to HTS. Because the SFB assay is a cell-based assay, the dependence on cell culture requires planning ahead to ensure a sufficient cell count, limiting application of the assay to HTS. Serological assays complement each other to provide a better diagnosis; the cPass and RDT assays, which allow HTS, could serve as the first round of screening, and the more sensitive SFB assay could provide confirmation and further investigation of borderline/discrepant samples. We are also currently developing an assay to detect all seven isotypes in one single test using different fluorophores to reduce the number of tests per sample. There might also be limited application of the SFB assays because of the dependence on flow cytometers. Although we used a large flow cytometer (LSR4, BD Biosciences) in this study, we are also developing

CellPress
OPEN ACCESS

an assay for portable flow cytometers, such as the Accuri (BD Biosciences), which could be deployed in small laboratory settings in places such as airports or at borders.

STAR★METHODS

Detailed methods are provided in the online version of this paper and include the following:

- KEY RESOURCES TABLE
- RESOURCE AVAILABILITY
  - Lead contact
  - Materials availability
  - Data and code availability
- EXPERIMENTAL MODEL AND SUBJECT DETAILS
  - Ethics
  - Plasma samples
- METHOD DETAILS
  - Generation of S protein-expressing cell line
  - S protein flow cytometry-based assay (SFB assay) for antibody detection
  - GenScript cPass Neutralization Antibody Detection kit
  - WondFo SARS-CoV-2 antibody Rapid diagnostic test (RDT)
- QUANTIFICATION AND STATISTICAL ANALYSIS
  - Quantification of S protein antibody by flow cytometry
  - Statistical analysis

SUPPLEMENTAL INFORMATION

Supplemental Information can be found online at https://doi.org/10.1016/j.xcrm.2021.100193.

ACKNOWLEDGMENTS

The authors would like to thank the study participants who donated their blood samples for this study. The authors also wish to thank Ding Ying and his team for help with patient recruitment, Dr. Danielle Anderson and her team at Duke-NUS for technical assistance, and Dr. Anis Larbi at the Singapore Immunology Network (SIgN), A*STAR for providing healthy donor samples. This work was supported by the Biomedical Research Council (BMRC), the A*ccelerate GAP-funded project (ACCL/19-GAP064-R20H-H) from the Agency for Science, Technology and Research (A*STAR), and the National Medical Research Council (NMRC) COVID-19 Research Fund (COVID19RF-001, COVID-19RF-007, and COVID-19RF-60).

AUTHOR CONTRIBUTIONS

Y.S.G. conceptualized the study, designed and conducted the experiments, analyzed the data, and wrote the manuscript. A.L.J. and J.-M.C. designed and conducted the experiments and analyzed the data. B.L. analyzed the data and wrote the manuscript. P.X.H. conducted the experiments. S.N.A., C.Y.-P.L., R.S.-L.C., B.E.Y., M.I.-C.C., P.A.T., S.K., S.P., S.-Y.T., L.J.S., Y.-S.L., D.C.L., and R.T.P.L. designed and supervised sample collection. B.W., C.Y.L., E.Z.X.N., and C.-I.W. provided reagents. L.F.P.N., R.T.P.L., and L.R. conceptualized the study and wrote the manuscript. All authors revised and approved the final version of the manuscript.

DECLARATION OF INTERESTS

A patent application for the SFB assay has been filed (Singapore patent 10202009679P: A Method Of Detecting Antibodies And Related Products).

Received: October 2, 2020
Revised: December 6, 2020
Accepted: January 12, 2021
Published: January 16, 2021

REFERENCES

1. WHO (2020). WHO Coronavirus Disease (COVID-19). https://covid19.who.int/.
2. Cohen, J., and Normile, D. (2020). New SARS-like virus in China triggers alarm. Science *367*, 234–235.
3. Wu, F., Zhao, S., Yu, B., Chen, Y.M., Wang, W., Song, Z.G., Hu, Y., Tao, Z.W., Tian, J.H., Pei, Y.Y., et al. (2020). A new coronavirus associated with human respiratory disease in China. Nature *579*, 265–269.
4. Zhou, P., Yang, X.L., Wang, X.G., Hu, B., Zhang, L., Zhang, W., Si, H.R., Zhu, Y., Li, B., Huang, C.L., et al. (2020). A pneumonia outbreak associated with a new coronavirus of probable bat origin. Nature *579*, 270–273.
5. Corman, V.M., Landt, O., Kaiser, M., Molenkamp, R., Meijer, A., Chu, D.K., Bleicker, T., Brünink, S., Schneider, J., Schmidt, M.L., et al. (2020). Detection of 2019 novel coronavirus (2019-nCoV) by real-time RT-PCR. Euro Surveill. *25*, 2000045.
6. Chu, D.K.W., Pan, Y., Cheng, S.M.S., Hui, K.P.Y., Krishnan, P., Liu, Y., Ng, D.Y.M., Wan, C.K.C., Yang, P., Wang, Q., et al. (2020). Molecular Diagnosis of a Novel Coronavirus (2019-nCoV) Causing an Outbreak of Pneumonia. Clin. Chem. *66*, 549–555.
7. Hartman, W.R., Hess, A.S., and Connor, J.P. (2020). Persistent viral RNA shedding after COVID-19 symptom resolution in older convalescent plasma donors. Transfusion, Published online June 13, 2020. https://doi.org/10.1111/trf.15927.
8. Pan, Y., Zhang, D., Yang, P., Poon, L.L.M., and Wang, Q. (2020). Viral load of SARS-CoV-2 in clinical samples. Lancet Infect. Dis. *20*, 411–412.
9. Zou, L., Ruan, F., Huang, M., Liang, L., Huang, H., Hong, Z., Yu, J., Kang, M., Song, Y., Xia, J., et al. (2020). SARS-CoV-2 Viral Load in Upper Respiratory Specimens of Infected Patients. N. Engl. J. Med. *382*, 1177–1179.
10. Lee, C.Y., Lin, R.T.P., Renia, L., and Ng, L.F.P. (2020). Serological Approaches for COVID-19: Epidemiologic Perspective on Surveillance and Control. Front. Immunol. *11*, 879.
11. Hahn, S.M. (2020). Coronavirus (COVID-19) Update: FDA Expedites Review of Diagnostic Tests to Combat COVID-19. https://www.fda.gov/news-events/press-announcements/coronavirus-covid-19-update-fda-expedites-review-diagnostic-tests-combat-covid-19.
12. FDA (2020). Coronavirus (COVID-19) Update: Daily Roundup August 17, 2020. https://www.fda.gov/news-events/press-announcements/coronavirus-covid-19-update-daily-roundup-august-17-2020.
13. Ahmed, S.F., Quadeer, A.A., and McKay, M.R. (2020). Preliminary Identification of Potential Vaccine Targets for the COVID-19 Coronavirus (SARS-CoV-2) Based on SARS-CoV Immunological Studies. Viruses *12*, 254.
14. Okba, N.M.A., Müller, M.A., Li, W., Wang, C., GeurtsvanKessel, C.H., Corman, V.M., Lamers, M.M., Sikkema, R.S., de Bruin, E., Chandler, F.D., et al. (2020). Severe Acute Respiratory Syndrome Coronavirus 2-Specific Antibody Responses in Coronavirus Disease Patients. Emerg. Infect. Dis. *26*, 1478–1488.
15. GeurtsvanKessel, C.H., Okba, N.M.A., Igloi, Z., Bogers, S., Embregts, C.W.E., Laksono, B.M., Leijten, L., Rokx, C., Rijnders, B., Rahamat-Langendoen, J., et al. (2020). An evaluation of COVID-19 serological assays informs future diagnostics and exposure assessment. Nat. Commun. *11*, 3436.
16. Hoffmann, M., Kleine-Weber, H., Schroeder, S., Krüger, N., Herrler, T., Erichsen, S., Schiergens, T.S., Herrler, G., Wu, N.H., Nitsche, A., et al. (2020). SARS-CoV-2 Cell Entry Depends on ACE2 and TMPRSS2 and Is Blocked by a Clinically Proven Protease Inhibitor. Cell *181*, 271–280.e8.

17. Lv, H., Wu, N.C., Tsang, O.T., Yuan, M., Perera, R., Leung, W.S., So, R.T.Y., Chan, J.M.C., Yip, G.K., Chik, T.S.H., et al. (2020). Cross-reactive antibody response between SARS-CoV-2 and SARS-CoV infections. bioRxiv. https://doi.org/10.1101/2020.03.15.993097.

18. Tai, W., He, L., Zhang, X., Pu, J., Voronin, D., Jiang, S., Zhou, Y., and Du, L. (2020). Characterization of the receptor-binding domain (RBD) of 2019 novel coronavirus: implication for development of RBD protein as a viral attachment inhibitor and vaccine. Cell. Mol. Immunol. *17*, 613–620.

19. Amanat, F., Stadlbauer, D., Strohmeier, S., Nguyen, T.H.O., Chromikova, V., McMahon, M., Jiang, K., Arunkumar, G.A., Jurczyszak, D., Polanco, J., et al. (2020). A serological assay to detect SARS-CoV-2 seroconversion in humans. Nat. Med. *26*, 1033–1036.

20. Wang, B., Asarnow, D., Lee, W.-H., Huang, C.-W., Faust, B., Ng, P.M.L., Ngoh, E.Z.X., Bohn, M., Bulkley, D., Pizzorno, A., et al. (2020). Bivalent binding of a fully human IgG to the SARS-CoV-2 spike proteins reveals mechanisms of potent neutralization. bioRxiv. https://doi.org/10.1101/2020.2007.2014.203414.

21. Amrun, S.N., Lee, C.Y., Lee, B., Fong, S.W., Young, B.E., Chee, R.S., Yeo, N.K., Torres-Ruesta, A., Carissimo, G., Poh, C.M., et al. (2020). Linear B-cell epitopes in the spike and nucleocapsid proteins as markers of SARS-CoV-2 exposure and disease severity. EBioMedicine *58*, 102911.

22. Ni, L., Ye, F., Cheng, M.L., Feng, Y., Deng, Y.Q., Zhao, H., Wei, P., Ge, J., Gou, M., Li, X., et al. (2020). Detection of SARS-CoV-2-Specific Humoral and Cellular Immunity in COVID-19 Convalescent Individuals. Immunity *52*, 971–977.e3.

23. Kawasaki, Y., Suzuki, J., Sakai, N., Isome, M., Nozawa, R., Tanji, M., and Suzuki, H. (2004). Evaluation of T helper-1/-2 balance on the basis of IgG subclasses and serum cytokines in children with glomerulonephritis. Am. J. Kidney Dis. *44*, 42–49.

24. Richardson, S.I., Lambson, B.E., Crowley, A.R., Bashirova, A., Scheepers, C., Garrett, N., Abdool Karim, S., Mkhize, N.N., Carrington, M., Ackerman, M.E., et al. (2019). IgG3 enhances neutralization potency and Fc effector function of an HIV V2-specific broadly neutralizing antibody. PLoS Pathog. *15*, e1008064.

25. Walker, M.R., Eltahla, A.A., Mina, M.M., Li, H., Lloyd, A.R., and Bull, R.A. (2020). Envelope-Specific IgG3 and IgG1 Responses Are Associated with Clearance of Acute Hepatitis C Virus Infection. Viruses *12*, 75.

26. Hofmeister, Y., Planitzer, C.B., Farcet, M.R., Teschner, W., Butterweck, H.A., Weber, A., Holzer, G.W., and Kreil, T.R. (2011). Human IgG subclasses: in vitro neutralization of and in vivo protection against West Nile virus. J. Virol. *85*, 1896–1899.

27. Bai, Y., Yao, L., Wei, T., Tian, F., Jin, D.Y., Chen, L., and Wang, M. (2020). Presumed Asymptomatic Carrier Transmission of COVID-19. JAMA *323*, 1406–1407.

28. Hu, Z., Song, C., Xu, C., Jin, G., Chen, Y., Xu, X., Ma, H., Chen, W., Lin, Y., Zheng, Y., et al. (2020). Clinical characteristics of 24 asymptomatic infections with COVID-19 screened among close contacts in Nanjing, China. Sci. China Life Sci. *63*, 706–711.

29. GenScript (2020). SARS-CoV-2 Surrogate Virus Neutralization Test (sVNT) Kit (RUO). https://www.genscript.com/covid-19-detection-svnt.html.

30. Bilcare Research (2020). Wondfo SARS-CoV-2 Antibody Test. https://www.bilcare.com/Wondfo/wondfo.html.

31. Long, Q.X., Tang, X.J., Shi, Q.L., Li, Q., Deng, H.J., Yuan, J., Hu, J.L., Xu, W., Zhang, Y., Lv, F.J., et al. (2020). Clinical and immunological assessment of asymptomatic SARS-CoV-2 infections. Nat. Med. *26*, 1200–1204.

32. Lieberman, M.M., Frank, W.J., and Brady, A.V. (1988). Protective mechanism of the immune response to a ribosomal vaccine from Pseudomonas aeruginosa. II. In vitro bactericidal and opsonophagocytic studies with specific antiserum. J. Surg. Res. *44*, 251–258.

33. Zollinger, W.D., and Mandrell, R.E. (1983). Importance of complement source in bactericidal activity of human antibody and murine monoclonal antibody to meningococcal group B polysaccharide. Infect. Immun. *40*, 257–264.

34. Taborda, C.P., Rivera, J., Zaragoza, O., and Casadevall, A. (2003). More is not necessarily better: prozone-like effects in passive immunization with IgG. J. Immunol. *170*, 3621–3630.

35. Goh, Y.S., Clare, S., Micoli, F., Saul, A., Mastroeni, P., and MacLennan, C.A. (2015). Monoclonal Antibodies of a Diverse Isotype Induced by an O-Antigen Glycoconjugate Vaccine Mediate In Vitro and In Vivo Killing of African Invasive Nontyphoidal Salmonella. Infect. Immun. *83*, 3722–3731.

36. Ibarrondo, F.J., Fulcher, J.A., Goodman-Meza, D., Elliott, J., Hofmann, C., Hausner, M.A., Ferbas, K.G., Tobin, N.H., Aldrovandi, G.M., and Yang, O.O. (2020). Rapid Decay of Anti-SARS-CoV-2 Antibodies in Persons with Mild Covid-19. N. Engl. J. Med. *383*, 1085–1087.

37. Seow, J., Graham, C., Merrick, B., Acors, S., Steel, K.J.A., Hemmings, O., O'Bryne, A., Kouphou, N., Pickering, S., Galao, R.P., et al. (2020). Longitudinal evaluation and decline of antibody responses in SARS-CoV-2 infection. Nat. Microbiol *5*, 1598–1607.

38. Chew, M.H., Koh, F.H., Wu, J.T., Ngaserin, S., Ng, A., Ong, B.C., and Lee, V.J. (2020). Clinical assessment of COVID-19 outbreak among migrant workers residing in a large dormitory in Singapore. J. Hosp. Infect. *106*, 202–203.

39. Grzelak, L., Temmam, S., Planchais, C., Demeret, C., Tondeur, L., Huon, C., Guivel-Benhassine, F., Staropoli, I., Chazal, M., Dufloo, J., et al. (2020). A comparison of four serological assays for detecting anti-SARS-CoV-2 antibodies in human serum samples from different populations. Sci. Transl. Med. *12*, eabc3103.

40. Singapore Statutes Online Plus (2003). Infectious Diseases Act. https://sso.agc.gov.sg/Act/IDA1976.

41. Pung, R., Chiew, C.J., Young, B.E., Chin, S., Chen, M.I., Clapham, H.E., Cook, A.R., Maurer-Stroh, S., Toh, M.P.H.S., Poh, C., et al.; Singapore 2019 Novel Coronavirus Outbreak Research Team (2020). Investigation of three clusters of COVID-19 in Singapore: implications for surveillance and response measures. Lancet *395*, 1039–1046.

42. Leong, H.N., Earnest, A., Lim, H.H., Chin, C.F., Tan, C., Puhaindran, M.E., Tan, A., Chen, M.I., and Leo, Y.S. (2006). SARS in Singapore–predictors of disease severity. Ann. Acad. Med. Singap. *35*, 326–331.

43. Jiang, L., Lee, V.J., Cui, L., Lin, R., Tan, C.L., Tan, L.W., Lim, W.Y., Leo, Y.S., Low, L., Hibberd, M., and Chen, M.I. (2017). Detection of viral respiratory pathogens in mild and severe acute respiratory infections in Singapore. Sci. Rep. *7*, 42963.

STAR★METHODS

KEY RESOURCES TABLE

REAGENT or RESOURCE	SOURCE	IDENTIFIER
**Antibodies**		
Anti-human IgG Alexa Fluor 647	Thermo Fisher Scientific	Cat# A21445; RRID:AB_2535862
Anti-human IgM Alexa Fluor 647	Thermo Fisher Scientific	Cat# A21249; RRID:AB_2535817
Anti-human IgA Alexa Fluor 647	BioLegend	Cat# 411502; RRID:AB_2650697
Anti-mouse IgG Alexa Fluor 647	Thermo Fisher Scientific	Cat# A21235; RRID:AB_2535804
Anti-human IgG1	Thermo Fisher Scientific	Cat# MA1-34581; RRID:AB_11004658
Anti-human IgG2	BioLegend	Cat# 411102; RRID:AB_2686940
Anti-human IgG3	BioLegend	Cat# 411302; RRID:AB_2686942
Anti-human IgG4	Thermo Fisher Scientific	Cat# A10651; RRID:AB_2534053
**Bacterial and virus strains**		
XL10 gold ultracompetent bacterial cells	Agilent	Cat# 200314
XL10 bacterial cells harboring pHIV-SARS-CoV-2-SP-eGPF	Generated in this study	NA
**Biological Samples**		
Plasma samples from symptomatic COVID-19 patients	N/A	IRB# 2020/00091
Plasma samples from recovered SARS patients	N/A	IRB# 2012/00917
Plasma samples from seasonal human CoV patients	N/A	IRB# 2020/00076
Plasma samples from healthy donors	N/A	IRB# 2017/2806 and IRB# 04-140
Plasma samples from National Public Health Laboratory	N/A	Singapore Infectious Diseases Act
**Chemicals, peptides, and recombinant proteins**		
EndoFectin Lenti	GeneCopoeia	Cat# EF001
Polybrene	Sigma-Aldrich	Cat# H9268
Dulbecco's Modified Eagle Medium (DMEM)	HyClone	Cat# SH30022.01
Fetal Bovine Serum (FBS)	HyClone	Cat# SV30160.03HI
Propidium Iodide	Sigma-Aldrich	Cat# P4170
ACE2-human Fc	Prof Wang Cheng-I's laboratory	NA
**Critical commercial assays**		
cPass Neutralization Antibody Detection kit	GenScript	Cat# L00847
SARS-CoV-2 antibody Rapid diagnostic test (RDT)	Guangzhou WondFo Biotech	Cat# W195
**Experimental Models: Cell Lines**		
HEK293T	ATCC	Cat# CRL-3216
HEK293T expressing full length S protein	Generated in this study	NA
**Oligonucleotides**		
EF1aFor (Table S2)	Integrated DNA Technologies	EF1aFor
SPseqF1 (Table S2)	Integrated DNA Technologies	SPseqF1
SPseqF2 (Table S2)	Integrated DNA Technologies	SPseqF2
SPseqF3 (Table S2)	Integrated DNA Technologies	SPseqF3
SPseqF4 (Table S2)	Integrated DNA Technologies	SPseqF4
SPseqR1 (Table S2)	Integrated DNA Technologies	SPseqR1
IRESrev (Table S2)	Integrated DNA Technologies	IRESrev
**Plasmids**		
pHIV-eGFP	Addgene	Cat# 21373
pMD2.G	Addgene	Cat# 12259

(*Continued on next page*)

Continued		
REAGENT or RESOURCE	SOURCE	IDENTIFIER
pMDLg/pRRE	Addgene	Cat# 12251
pRSV-Rev	Addgene	Cat# 12253
pHIV-SARS-CoV-2-SP-eGPF	Generated in this study	NA
Others		
BD Vacutainer® CPT tubes	BD Biosciences	Cat# 362753
96 V-bottomed well plates	Thermo Fisher Scientific	Cat# 249570

RESOURCE AVAILABILITY

Lead contact

Further information and requests for resources and reagents should be directed to and will be fulfilled by the Lead Contact: Laurent Renia, Infectious Diseases Laboratories (ID Labs) and Singapore Immunology Network-BMSI-A*STAR, 8A Biomedical Grove, #03-15, Immunos Building, Biopolis, Singapore 138648; Tel: +65 64070005; Fax: +65 6464 2056; Email: renia_laurent@immunol.a-star.edu.sg

Materials availability

All unique/stable reagents generated in this study are available from the Lead Contact with a completed Materials Transfer Agreement.

Data and code availability

This study did not generate any datasets/code.

EXPERIMENTAL MODEL AND SUBJECT DETAILS

Ethics

The study design and protocols for COVID-19, recovered SARS and seasonal human CoV patient cohorts were approved by National Healthcare Group (NHG) Domain Specific Review Board (DSRB) and performed, following ethical guidelines in the approved studies 2012/00917, 2020/00091 and 2020/00076 respectively. Healthy donor samples were collected in accordance with approved studies 2017/2806 and NUS IRB 04-140. Written informed consent was obtained from participants in accordance with the Declaration of Helsinki for Human Research.

All samples received at the National Public Health Laboratory (NPHL) were collected under Singapore Infectious Diseases Act, which allows epidemiological studies and use of data for analysis to control outbreaks.[40]

Plasma samples

COVID-19 patients

A total of 81 patients (Table S1), who were tested PCR-positive for SARS-CoV-2 in the nasopharyngeal swab, were recruited into the study from January to March 2020.[41] Demographic data, clinical and laboratory parameters during the hospitalisation period were retrieved from patient records (Table S1).[21] Whole blood of patients was collected into BD Vacutainer® CPT tubes and centrifuged at 1700 g for 20 min to obtain plasma fractions. Plasma samples were categorised according to three time points: median 5 days post-illness onset (pio), median 10 days pio, and median 23 days pio.

Recovered SARS and seasonal human CoV patients

A total of 20 individuals (Table S1) previously diagnosed with SARS-CoV during the outbreak in 2003[42] were contacted and enrolled. Plasma fractions were isolated from recovered SARS individuals described above. Archived samples from human CoV patients (Table S1) collected between 2012-2013 were also used in this study. This included post-infected samples from seven alpha-CoV (229E/NL63) and six beta-CoV (OC43) infections confirmed using the SeeGene RV12 respiratory multiplex kit.[43]

Samples received at NPHL

Plasma samples received at the National Public Health Laboratory (NPHL) (Table S1) were collected from convalescent cases, suspected infections and general populations for sero-prevalence studies. A total of 109 samples was categorised based on PCR-status and patient symptoms status (Table S1): PCR-positive and symptomatic, n = 16, PCR-positive and pre-/asymptomatic, n = 34, PCR-positive and patient symptom status-unknown, n = 11, PCR-negative and patient symptom status-unknown, n = 13, PCR status-negative/not done and no symptom, n = 20, PCR status-unknown and patient symptom status-unknown, n = 15. Patients were

defined as symptomatic if there is presence of one of following symptoms: fever, runny nose, sore throat, cough, anosmia, dysgeusia or breathlessness.

METHOD DETAILS

Generation of S protein-expressing cell line

The SARS-Cov-2 S gene (GenBank: QHD43416.1), which encodes for the S protein, was codon-optimized (Table S2) for expression by human mammalian cells. Full length S gene was cloned into pHIV-eGFP transfer plasmid, via the XbaI and BamHI sites, upstream of an IRES (internal ribosome entry site) and a eGFP gene (Table S2). The transfer plasmid, pHIV-SARS-CoV-2-SP-eGPF, was then co-transfected with the packaging and envelope plasmids (pMD2.G, pMDLg/pRRE and pRSV-Rev) into HEK293T cells using Endo-Fectin Lenti. The medium (DMEM + 10% FBS) was changed 8-16 h later and the lentiviral particles in the supernatant were collected after a further 48 h incubation. Cells were transduced by adding the lentiviral supernatant and 8 μg/ml polybrene, then centrifuging at 1200 x g for 1 h at room temperature. The medium was changed after 8-16 h in the cell culture incubator. After a further 48 h incubation, eGFP-expressing HEK293T cells were sorted, expanded and cryopreserved.

Expression of S protein was confirmed by ACE2 binding (Figure S1). Briefly, cells were seeded at 1.5×10^5 cells per well in 96 well V-bottomed plates. The cells were first incubated with ACE2-HuFc (ACE2 protein tagged with a human Fc region, 6.25 μg/ml) before a secondary incubation with a double stain, consisting of Alexa Fluor 647-conjugated anti-human IgG (diluted 1:500) and propidium iodide (PI; diluted 1:2500). Cells were read on LSR4 laser (BD Biosciences) and analyzed using FlowJo (Tree Star).

S protein flow cytometry-based assay (SFB assay) for antibody detection

S protein-expressing cells were seeded at 1.5×10^5 cells per well in 96 well V-bottom plates. The cells were first incubated with human serum (diluted 1:100 in 10% FBS) before a secondary incubation with a double stain, consisting of Alexa Fluor 647-conjugated secondary antibodies (diluted 1:500) and propidium iodide (PI; diluted 1:2500). Secondary antibodies used are conjugated anti-human IgM, or IgG. For assays examining IgG subclasses, the secondary incubation was with mouse anti-human IgG1, IgG2, IgG3, or anti-human IgG4. Following the secondary incubation, the cells were then incubated with Alexa Fluor 647-conjugated anti-mouse IgG. Cells were read on BD Biosciences LSR4 laser and analyzed using FlowJo (Tree Star).

GenScript cPass Neutralization Antibody Detection kit

Serum samples were analyzed by the GenScript cPass Neutralization Antibody Detection kit, according to the manufacturer's instructions. Briefly, the serum samples were first diluted 1:10 in provided sample dilution buffer and then mixed with HRP-conjugated RBD with a volume ratio of 1:1 and incubated at 37°C for 30 min. The mixture was added to wells in the capture plate in the kit for another incubation at 37°C for 15 min. After washing, TMB solution was added to the plate and the plate was incubated in the dark for 15 min at 25°C. Absorbance at 450 nm was read immediately with Sunrise Microplate Reader (Tecan) after the addition of the stop solution. Samples were defined as positive when the inhibition is $\geq$ 20% - inhibition was calculated as Inhibition = (1 - OD value of Sample/OD value of Negative Control) × 100%, according to manufacturer's instructions. Reading of 10%–30% inhibition was defined as borderline results.

WondFo SARS-CoV-2 antibody Rapid diagnostic test (RDT)

Serum samples were analyzed by the WondFo SARS-CoV-2 antibody RDT according to the manufacturer's instructions. Briefly, serum samples were first added to the sample wells before the addition of the detection buffer into the buffer well. The test kit was then left at room temperature for 15 min before being visually read.

QUANTIFICATION AND STATISTICAL ANALYSIS

Quantification of S protein antibody by flow cytometry

Binding of specific antibody binding to cells were determined by LSR4 laser (BD Biosciences) and analyzed using FlowJo (Tree Star). Cells were gated on: FSC-A/SSC-A to exclude cell debris (Figure S1A), FSC-A/FSC-H to select for single cells (Figure S1B), FSC-A/PI to select for live cells (PI-negative population; Figure S1C), FITC/Alexa Fluor 647 (Figures S1D–S1H). Binding is determined by the percentage of GFP-positive S protein-expressing cells that are bound by specific antibody, indicated by the events that are Alexa Fluor 647- and FITC-positive (Gate 2). A sample is defined as positive when the binding is more than mean + 3SD of the healthy controls (n = 22). The thresholds using the healthy control readings is based on the normal-like distribution of the healthy control reading where a mean + 3SD threshold would mean that there is less than a 0.13% chance of a false positive. Receiver Operating Characteristic (ROC) curves were constructed from each of the antibody binding with the healthy controls and SARS-CoV-2 patients as the true negatives and true positives respectively using the pROC library in R version 3.6.4.

Statistical analysis

Statistical analysis was done using GraphPad Prism (GraphPad Software). For comparing between multiple groups, Kruskal-Wallis tests and post hoc tests using Dunn's multiple comparison tests were used to identify significant differences. For paired comparison

between total IgG and IgG1 response, Wilcoxon matched-pairs signed rank test was used. P values less than 0.05 are considered significant, where * indicates $p \leq 0.05$, ** indicates $p \leq 0.01$, *** indicates $p \leq 0.001$, **** indicates $p \leq 0.0001$.

Receiver Operating Characteristic (ROC) curves were constructed from each of the antibody binding with the healthy controls and SARS-CoV-2 patients as the true negatives and true positives respectively using the pROC library in R version 3.6.4.

Cell Reports Medicine, Volume *2*

Supplemental Information

Sensitive detection of total anti-Spike antibodies

and isotype switching in asymptomatic

and symptomatic individuals with COVID-19

Yun Shan Goh, Jean-Marc Chavatte, Alicia Lim Jieling, Bernett Lee, Pei Xiang Hor, Siti Naqiah Amrun, Cheryl Yi-Pin Lee, Rhonda Sin-Ling Chee, Bei Wang, Chia Yin Lee, Eve Zhi Xian Ngoh, Cheng-I Wang, Barnaby Edward Young, Paul A. Tambyah, Shirin Kalimuddin, Surinder Pada, Seow-Yen Tan, Louisa Jin Sun, Mark I-Cheng Chen, Yee-Sin Leo, David C. Lye, Lisa F.P. Ng, Raymond Tzer Pin Lin, and Laurent Renia

Sensitive detection of total anti-Spike antibodies and isotype switching in asymptomatic and symptomatic COVID-19 patients

Yun Shan Goh[1,2], Jean-Marc Chavatte[3], Alicia Lim Jieling[3], Bernett Lee[2], Pei Xiang Hor[1,2], Siti Naqiah Amrun[1,2], Cheryl Yi-Pin Lee[1,2], Rhonda Sin-Ling Chee[1,2], Bei Wang[2], Chia Yin Lee[2], Eve Zhi Xian Ngoh[2], Cheng-I Wang[2], Barnaby Edward Young[3,4,5], Paul A. Tambyah[3,6], Shirin Kalimuddin[7,8], Surinder Pada[9], Seow-Yen Tan[10], Louisa Jin Sun[11], Mark I-Cheng Chen[3,12], Yee-Sin Leo[3,4,5,13], David C. Lye[3,4,5,13], Lisa F.P. Ng[1,2,14,15,16,17], Raymond Tzer Pin Lin[3,13,18], Laurent Renia[1,2,17,18,19,*]

[1]Infectious Diseases Laboratories (ID Labs), Agency for Science, Technology and Research (A*STAR), Immunos, Biopolis, Singapore 138648, Singapore.

[2]Singapore Immunology Network, Agency for Science, Technology and Research (A*STAR), Immunos, Biopolis, Singapore 138648, Singapore.

[3]National Centre for Infectious Diseases, 16 Jalan Tan Tock Seng, Singapore 308442, Singapore.

[4]Department of Infectious Diseases, Tan Tock Seng Hospital, 11 Jalan Tan Tock Seng, Singapore 308433, Singapore.

[5]Lee Kong Chian School of Medicine, Nanyang Technological University, 11 Mandalay
20 Road, Singapore 308232, Singapore.

[6]Department of Medicine, National University Hospital, 5 Lower Kent Ridge Road, Singapore 119074, Singapore.

[7]Department of Infectious Diseases, Singapore General Hospital, 31 Third Hospital
Ave, Singapore 168753, Singapore.

[8]Emerging Infectious Disease Program, Duke-NUS Medical School, 8 College Road, Singapore 169857, Singapore.

[9]Division of Infectious Diseases, Ng Teng Fong Hospital, 1 Jurong East Street 21, Singapore 609606, Singapore.

[10]Department of Infectious Diseases, Changi General Hospital, 2 Simei Street 3, Singapore 529889, Singapore.

[11]Alexandra Hospital, 378 Alexandra Road, Singapore 159964, Singapore.

[12]Saw Swee Hock School of Public Health, National University of Singapore and National University Health System, 12 Science Drive 2, Singapore 117549, Singapore

[13]Yong Loo Lin School of Medicine, National University of Singapore and National University Health System, 10 Medical Drive, Singapore 117597, Singapore.

[14]Department of Biochemistry, Yong Loo Lin School of Medicine, National University of Singapore, Singapore 117596, Singapore.

[15]National Institute of Health Research, Health Protection Research Unit in Emerging and Zoonotic Infections, University of Liverpool, Liverpool, United Kingdom.

[16]Institute of Infection, Veterinary and Ecological Sciences, University of Liverpool, Liverpool, United Kingdom.

[17]Department of Microbiology and Immunology, Yong Loo Lin School of Medicine, National University of Singapore, Singapore 117597, Singapore.

[18]Senior author

[19]Lead contact

***Corresponding author**: Laurent Renia, A* STAR Infectious Diseases Laboratories (ID Labs), 8A Biomedical Grove, #03-15, Immunos Building, Biopolis, Singapore 138648; Tel: +65 64070005; Fax: +65 6464 2056; Email: renia_laurent@immunol.a-star.edu.sg

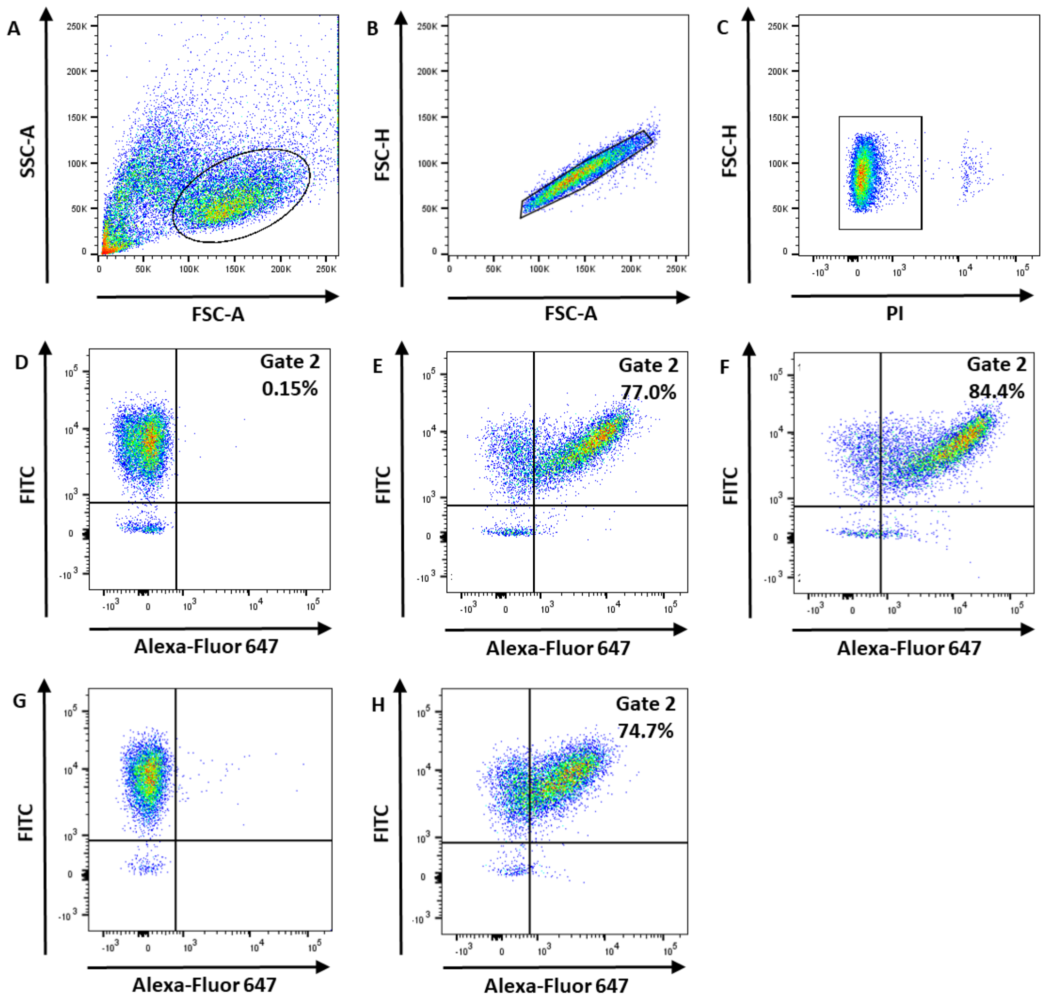

Figure S1. FACS plot analysis. Cells were gated on: (A) FSC-A/SSC-A to exclude cell debris, (B) FSC-A/FSC-H to select for single cells, (C) FSC-A/PI to select for live cells (PI-negative population), (D, E, F, G, H) FITC/Alexa Fluor 647. Binding is determined by the percentage of GFP-positive S protein-expressing cells that are bound by specific antibody, indicated by the events that are Alexa Fluor 647- and FITC-positive (Gate 2). (D) PBS control; (E) 6.25 µg/ml ACE-Human Fc; (F) 1 µg/ml 5A6 S protein RBD-specific monoclonal antibody; (G) healthy control plasma, 1:100 diluted; (H) COVID-19 patient plasma, 1:100 diluted. Related to Figure 1.

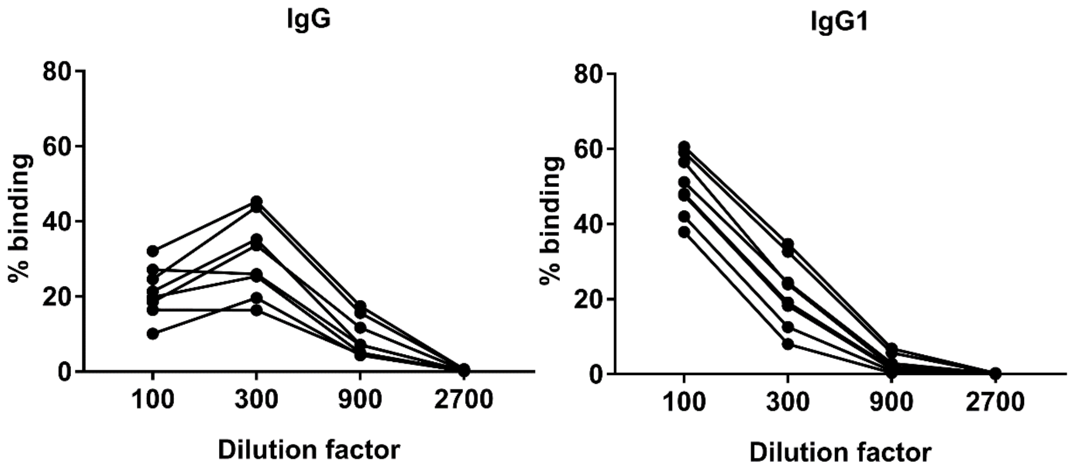

Figure S2. Effect of sample dilution on total IgG and IgG1 response. A total of eight samples from the NPHL cohort, where higher IgG1 response was observed over total IgG at dilution factor 100, were further analysed. The total IgG and IgG1 response were analysed by the SFB assay over four dilution factors (100, 300, 900, 2700). Data are shown as mean of two independent experiments. Related to Figure 4.

Table S1. Demographic and clinical information of symptomatic COVID-19 patients, healthy donors, recovered SARS, seasonal human CoV patients, and samples from National Public Health laboratory (NPHL), Related to STAR Methods Section

**Symptomatic COVID-19 patients**	**Patients (N=81)**
Age, Mean years (SD)	45 (13)
Gender, n (%)	
Male	48 (59.3%)
Female	33 (40.7%)
Ethnicity, n (%)	
Chinese	68 (84.0%)
Others	13 (16.0%)
Co-morbidities, n (%)	
Diabetes	7 (8.6%)
Hypertension	15 (18.5%)
Others	11 (13.6%)
Vital signs at admission	
Temperature, Mean °C, (SD)	37.7 (0.9)
Heart rate, Mean beats/minute (SD)	91.4 (16.6)
Respiratory rate, Mean rate per minute (SD)	18.4 (1.9)
Diastolic blood pressure, Mean mmHg (SD)	97.5 (2.4)
Systolic blood pressure, Mean mmHg (SD)	132.2 (18.5)
Oxygen saturation, Mean % (SD)	77.8 (15.2)
Laboratory findings	
Haemoglobin, Mean g/dL (SD)	13.8 (1.6)
Haematocrit, Mean % (SD)	40.8 (4.6)
Platelets, Mean x 10^9/L (SD)	194.8 (69.8)
White blood cells, Mean x 10^9/L (SD)	5.3 (3.0)
Lymphocytes, Mean x 10^9/L (SD)	1.2 (0.6)
Neutrophils, Mean x 10^9/L (SD)	4.3 (7.7)
Monocytes, Mean x 10^9/L (SD)	0.6 (1.1)
C-reactive protein, Mean mg/L (SD)	37.4 (55.7)
Creatinine, Mean μmol/L (SD)	75.0 (45.3)
Lactate dehydrogenase, Mean U/L (SD)	514.3 (298.4)
Alanine aminotransferase, Mean U/L (SD)	34.6 (28.1)
**Healthy donors**	**N=22***
Age, Mean years (SD)	45 (13)
Gender, n (%)	
Male	9 (40.9%)
Female	12 (54.5%)
**Recovered SARS**	**N=20**
Age, Mean years (SD)	48 (13)
Gender, n (%)	
Male	5 (25.0%)
Female	15 (75.0%)
**Seasonal human CoV**	**N=20**
Age, Mean years (SD)	44 (16)
Gender, n (%)	
Male	7 (53.8%)
Female	6 (46.2%)
**NPHL Samples**	**N=108**
**PCR-positive**	
**Symptomatic**	**16**
Convalescent COVID-19 patients	14/16

Random testing of general population	2/16
**No symptom**	**34**
MOH** surveillance of dormitory residents	33/34
MOH surveillance of suspected and quarantined cases	1/34
**Symptom-unknown**	**11**
MOH surveillance of suspected and quarantined cases	11/11
**PCR-negative**	
**Symptom-unknown**	**13**
MOH surveillance of suspected and quarantined cases	13/13
**PCR-negative/not done**	
**No Symptom**	**20**
MOH surveillance of dormitory residents	20/20
**PCR-unknown**	
**Symptom-unknown**	**15**
MOH surveillance of suspected and quarantined cases	9/15
Random testing of general population	6/15

* Information of one donor unknown. **Ministry of Health. SARS: Severe Acute Respiratory Syndrome Coronavirus; human CoV: human coronavirus

Table S2. DNA sequence of codon-optimised SARS-CoV-2 S gene and primers used to sequence full length SARS-CoV-2-S protein, Related to STAR Methods Section

SARS-CoV-2 S gene Codon-optimised	ATGTTTGTATTCTTGGTACTTCTCCCATTGGTATCTTCTCAATGCGTTAACCTTACCACACGC<br>ACCCAACTGCCCCCGGCCTACACTAATAGCTTTACGCGGGGTGTCTACTATCCCGACAAAGT<br>CTTTCGATCCAGTGTGCTCCACTCCACCCAGGATCTTTTCCTTCCCTTTTTTTCTAATGTTAC<br>GTGGTTCCACGCAATCCATGTATCCGGTACGAATGGGACAAAACGCTTTGACAATCCAGTG<br>CTGCCATTTAATGATGGAGTGTACTTTGCATCTACCGAGAAGAGTAACATCATCAGAGGAT<br>GGATCTTCGGAACGACCTTGGACTCCAAAACGCAATCCTTGCTTATCGTTAACAATGCAAC<br>GAATGTTGTCATCAAAGTTTGCGAATTCCAATTCTGTAACGATCCCTTCCTCGGTGTTTATTA<br>TCATAAAAATAATAAATCTTGGATGGAAAGTGAGTTCCGCGTATACAGTTCCGCCAATAAT<br>TGTACCTTCGAATACGTAAGTCAACCGTTCTTGATGGATCTGGAAGGTAAACAGGGTAACT<br>TTAAGAACCTTCGGGAGTTTGTTTTTAAGAACATAGACGGCTACTTTAAGATCTATAGTAAA<br>CATACGCCAATTAACTTGGTTAGAGATCTCCCGCAGGGGTTTTCAGCATTGGAGCCGCTCGT<br>CGACCTCCCCATAGGTATAAATATAACTCGGTTTCAAACACTGCTGGCGCTCCACCGCAGCT<br>ACCTGACGCCTGGGGATTCTTCTTCCGGTTGGACTGCAGGCGCTGCTGCATATTATGTAGGG<br>TACCTGCAACCGAGAACCTTTCTCCTTAAGTACAACGAGAATGGCACTATTACGGACGCTG<br>TCGATTGTGCACTCGACCCCTTGAGTGAGACGAAGTGTACACTGAAAAGCTTTACTGTTGA<br>AAAGGGAATATATCAGACATCCAACTTTAGAGTTCAGCCAACAGAATCCATCGTTCGATTT<br>CCCAATATTACAAATCTCTGTCCGTTCGGAGAGGTCTTTAATGCTACCCGATTCGCGTCAGT<br>ATACGCCTGGAACAGAAAGAGAATTTCTAACTGTGTTGCAGATTATAGTGTCCTGTATAATT<br>CTGCGTCTTTTAGCACTTTTAAGTGCTACGGCGTTAGCCCCACTAAGTTGAACGACCTTTGT<br>TTCACTAACGTGTATGCCGACTCATTCGTCATAAGAGGCGACGAAGTTAGACAAATTGCAC<br>CGGGCCAGACGGGAAAGATTGCGGACTACAACTATAAATTGCCTGACGACTTTACAGGATG<br>TGTCATCGCCTGGAATAGTAATAACCTTGACTCCAAAGTCGGTGGCAATTACAATTACTTGT<br>ACCGGCTGTTCAGGAAGTCTAATCTCAAACCTTTTGAGCGAGATATCAGCACGGAAATTTA<br>TCAAGCTGGTAGCACTCCATGTAACGGGGTTGAGGGTTTTAATTGTTATTTTCCATTGCAAT<br>CATATGGATTCCAACCGACTAACGGTGTTGGGTATCAACCATACAGAGTGGTGGTTTTGTCA<br>TTTGAACTTCTGCATGCCCCTGCAACAGTGTGCGGACCGAAGAAGAGTACGAACCTTGTAA<br>AGAACAAGTGCGTCAACTTCAACTTTAATGGTCTGACGGGTACCGGCGTTCTGACGGAATC<br>CAATAAAAAGTTCTTGCCCTTTCAGCAGTTCGGGCGAGATATCGCCGACACTACTGATGCG<br>GTGCGAGATCCTCAGACACTTGAGATCCTCGATATTACCCCATGTAGTTTTGGTGGTGTGTC<br>TGTGATTACACCCGGCACCAATACGTCAAATCAGGTCGCAGTCTTGTACCAAGACGTGAAC<br>TGCACCGAAGTTCCTGTAGCCATTCACGCTGATCAATTGACACCGACATGGAGGGTGTACT<br>CCACCGGATCTAACGTGTTCCAGACCCGCGCGGGGTGTCTTATCGGCGCAGAACATGTGAA<br>CAACTCTTACGAATGTGATATTCCTATCGGTGCAGGCATCTGTGCCTCATACCAGACACAAA<br>CGAACTCACCAAGGAGGGCAAGGTCAGTAGCCTCACAAAGCATAATAGCCTATACGATGA<br>GTCTTGGTGCGGAGAACTCTGTGGCGTACTCTAATAACTCTATCGCCATACCGACTAACTTC<br>ACCATTTCTGTTACGACCGAGATCCTCCCAGTTTCCATGACTAAGACAAGTGTGGATTGTAC<br>AATGTACATCTGCGGCGACAGTACTGAGTGCAGTAACCTGCTTCTGCAGTACGGGTCCTTCT<br>GCACACAACTTAACCGGGCGCTGACTGGTATAGCGGTTGAACAAGACAAGAACACTCAAG<br>AGGTCTTCGCACAAGTAAAACAAATATACAAAACACCACCTATTAAAGATTTCGGCGGGTT<br>TAATTTTAGCCAAATCCTTCCAGACCCCAGCAAACCCTCTAAGCGCAGCTTCATTGAGGATC<br>TGCTGTTTAACAAGGTCACCCTGGCAGACGCGGGCTTTATCAAGCAATACGGTGACTGCCT<br>GGGGGATATCGCGGCTCGAGACCTTATATGTGCGCAAAAATTTAATGGACTTACCGTACTT<br>CCTCCATTGCTGACTGACGAGATGATAGCACAGTATACATCTGCACTGCTCGCCGGTACAAT<br>TACATCAGGGTGGACATTTGGGGCGGGAGCTGCGCTCCAGATACCGTTCGCGATGCAGATG<br>GCGTATAGGTTTAATGGAATTGGTGTCACGCAAAACGTTCTCTATGAAAACCAGAAGCTGA<br>TAGCAAATCAGTTCAATTCCGCGATTGGTAAGATACAAGATTCATTGTCTAGTACGGCCTCT<br>GCACTCGGAAAACTCCAAGATGTAGTGAACCAAAACGCCCAAGCCCTGAATACACTCGTAA<br>AACAGCTCTCTAGTAATTTTGGGGCCATTTCCTCCGTATTGAACGACATCTTGAGTCGCTTG<br>GATAAGGTAGAAGCAGAAGTACAAATTGACCGGTTGATCACGGGCAGACTTCAATCACTTC<br>AGACTTATGTTACTCAGCAGCTTATACGAGCTGCAGAAATTCGCGCCTCTGCGAACCTGGCC<br>GCCACTAAAATGTCAGAATGTGTACTGGGACAGAGCAAACGGGTGGATTTCTGCGGAAAG<br>GGCTATCATCTGATGAGTTTTCCCCAGTCTGCGCCTCATGGTGTAGTATTTCTTCATGTCACA<br>TATGTACCAGCCCAAGAAAAAAATTTCACAACGGCGCCCGCGATTTGCCATGACGGTAAGG<br>CGCATTTTCCTCGCGAGGGCGTTTTCGTGTCTAACGGTACTCACTGGTTCGTAACACAGCGA<br>AACTTTTACGAGCCTCAGATAATCACGACGGATAACACATTTGTCTCCGGCAACTGCGATGT<br>GGTCATCGGTATAGTGAACAATACGGTATATGATCCGCTGCAGCCAGAGCTCGACAGTTTC<br>AAGGAGGAGCTTGACAAATACTTTAAGAACCATACCTCCCCAGACGTAGACCTCGGAGACA

	TATCTGGTATCAATGCCTCCGTGGTTAACATACAAAAGGAGATAGATAGACTGAATGAGGT GGCGAAGAATCTGAATGAGTCTCTCATAGATCTGCAGGAACTCGGTAAATATGAACAATAC ATCAAGTGGCCTTGGTACATCTGGCTGGGGTTCATAGCGGGCCTGATCGCGATCGTGATGG TAACTATAATGTTGTGTTGCATGACCTCCTGCTGCTCATGCCTTAAAGGTTGTTGTTCTTGCG GGAGCTGCTGCAAGTTCGATGAGGATGATTCAGAACCCGTCTTGAAGGGCGTAAAACTTCA CTATACGTAA	
Primers used to sequence full length SARS-CoV-2-S protein	EF1aFor	GGATCTTGGTTCATTCTCAAG
	SPseqF1	GTACCTGCAACCGAGAAC
	SPseqF2	GGCGTTCTGACGGAATC
	SPseqF3	GCAATACGGTGACTGCC
	SPseqF4	CGTGTCTAACGGTACTCAC
	SPseqR1	GTTCTCGGTTGCAGGTAC
	IRESrev	CATATAGACAAACGCACACC
